# Light-triggered modulation in donor–acceptor dipeptide assemblies

**DOI:** 10.1039/d6cc03505a

**Published:** 2026-07-13

**Authors:** Dipankar Ghosh, Ravi R. Sonani, Chengcheng Zhao, Simona Bianco, Edward H. Egelman, Duncan H. Gregory, Dave J. Adams

**Affiliations:** a School of Chemistry, University of Glasgow Glasgow G12 8QQ UK Dave.Adams@glasgow.ac.uk; b Department of Biochemistry and Molecular Genetics, University of Virginia Charlottesville VA 22903 USA; c MAX IV Laboratory, Lund University 224 84 Lund Sweden

## Abstract

Electron-deficient and electron-rich naphthalene-capped dipeptides 1NO_2_FF and 6OMeVF form co-assembled structure driven by complementary interactions. Light irradiation induces a reversible nanoscale structural modulation.

## Abstract 2

Electron-rich and electron-deficient naphthalene-capped dipeptides co-assemble into a light-responsive network that shows nanoscale reorganisation and a reversible yellow–to–red response.

## Graphical abstract

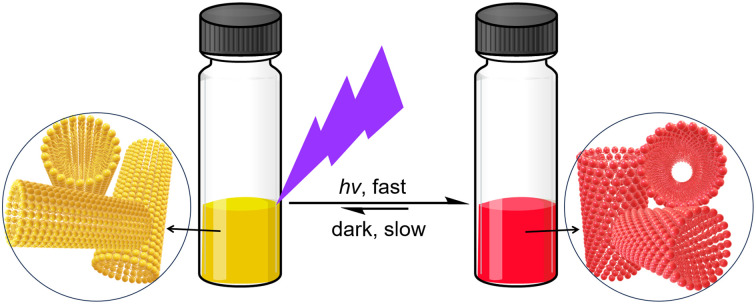

Multicomponent low-molecular-weight gelators enable the assembly of functional soft materials beyond the individual gelators.^1–3^ The resulting systems can generate distinct network structures that allow modular formulation, compositional gradients, and tuneable mechanical behaviour without changing the underlying chemistry.^2,4–6^ Mixtures may self-sort or form mixed assembled states, and small changes in concentration, mixing process, solvent system, ionic strength, or temperature can shift the balance between these behaviours.^1,7–11^ Achieving controllable and reversible switching between distinct assembled states is particularly difficult, since many systems become kinetically trapped.^12^

Dipeptide gelators provide a useful platform to explore these challenges. They are straightforward to synthesise, readily tuned through sequence and *N*-terminal capping group, and form assemblies in aqueous or mixed solvents.^7,13,14^ A further advantage is their tendency to generate anisotropic morphologies, including tapes, ribbons, and nanotubes.^13,15^ These assemblies are attractive because they can mimic biological filaments, act as nanoscale templates, and introduce directional mechanical responses within soft materials.^16–18^ In multicomponent systems, such morphologies may also provide routes by which one component influences the packing and growth of another, amplifying small molecular differences into distinct assembled structures.^10,19^

Multicomponent assembly can be directed through donor–acceptor (D–A) complementarity, as electron-rich and electron-poor aromatic units favour mixed packing.^20–23^ These interactions may bias assembly towards mixed states over self-sorting, alter fibre cohesion, and influence optical or electronic properties.^20–23^ Previous studies have used D–A pairs to influence morphology, tune emission, or improve charge transport in supramolecular materials.^20–23^ However, most systems have been designed to form a single preferred assembled structure, and comparatively little attention has been paid to how external stimuli can modify these interactions and alter aggregate-state organisation.^12,24,25^

Here, we use a naphthalene-capped dipeptide scaffold as a modular basis for a two-component system in which D–A complementarity promotes mixed aromatic interactions. Pairing two gelators with a common aromatic motif but different electronic character enables access to light-responsive assembled states while maintaining the same overall formulation. Beyond improving understanding of assembly behaviour in multicomponent dipeptide systems, this approach enables the design of optically responsive soft materials and sensing platforms.^4,5,14^

Using this approach, we prepared a multicomponent system (Fig. 1a and Fig. S1–S6) consisting of an electron-deficient aromatic gelator, 1NO_2_-2NapFF (1NO_2_FF), and an electron-rich analogue, 6OMe-2NapVF (6OMeVF). Stock solutions were prepared by stirring each compound with 1 equiv. aqueous NaOH at 10 mM (∼5 mg mL^−1^). Under these conditions, 1NO_2_FF formed a moderately viscous yellow solution (Fig. 1b), whereas 6OMeVF remained colourless with low viscosity. An equimolar mixture of 1NO_2_FF and 6OMeVF (Fig. 1b) was visually similar to the 1NO_2_FF solution. To rule out dilution effect, a higher-concentration equimolar mixture (10 mM each) was compared with 10 mM 1NO_2_FF, indicating that addition of 6OMeVF produced little change in colour under ambient conditions (Fig. S7).

**Fig. 1:**
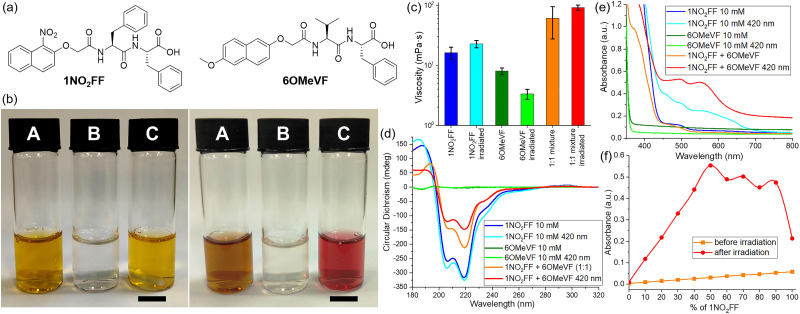
(a) Chemical structures of 1NO_2_FF and 6OMeVF. (b) Photographs of solutions before (left) and after (right) irradiation at 420 nm (30 min): [A] 1NO_2_FF (10 mM); [B] 6OMeVF (10 mM); [C] equimolar mixture of 1NO_2_FF and 6OMeVF (5 mM each). Scale bars = 1 cm. (c) Viscosity at a shear rate of 1 s^−1^ for the same samples before and after 420 nm irradiation. (d) CD spectra for the same samples before and after irradiation, corresponding HT spectra are shown in Fig. S13. (e) UV-vis absorption spectra of the single- and multi-component mixtures, and (f) Job's plot of absorbance at 550 nm for 1NO_2_FF/6OMeVF mixtures with different molar ratios before and after 420 nm irradiation for 30 min.

To evaluate the stimuli-responsiveness, we examined the effect of heat and light on the multicomponent system. Thermal stimulation was ineffective, as heating the mixture at 75 °C for 2 h produced no visible change (Fig. S8). In contrast, under sunlight, both the 1NO_2_FF and multicomponent solutions darkened, changing from yellow to brown and red, respectively, indicating light-triggered structural modulation. Such yellow-to-orange/red colour changes are associated with increased D–A interactions that generate lower-energy visible absorption features.^26–29^ To improve reproducibility, subsequent experiments were carried out using a solar simulator, which produced the same transformation. The effective excitation window was then investigated using monochromatic LEDs at 420, 455, 470, 505, and 565 nm to irradiate for 30 min (Fig. S9). Only 420 nm irradiation produced a pronounced colour change in the 1NO_2_FF and multicomponent systems (Fig. 1b and Fig. S10); other wavelengths produced no visible response. This wavelength dependence suggests excitation of a specific electronic transition associated with the assembled structures.^20–22^ When the irradiated 1NO_2_FF and multicomponent samples were stored in the dark, the original colour gradually recovered over ∼14 d (Fig. S11), indicating that the optical effect is reversible. The slow recovery may reflect retention of favourable D–A interactions in the light-adapted network.^30,31^

The bulk flow behaviour of the solutions was assessed by steady-shear viscosity measurements. 6OMeVF displayed viscosities close to the water baseline (∼10^−3^ Pa s)^32^ at both 10 and 5 mM concentrations with only weak shear dependence (Fig. 1c and Fig. S12), consistent with relatively short supramolecular assemblies.^33^ In contrast, 1NO_2_FF showed higher viscosity and mild shear thinning, indicating the presence of elongated shear-sensitive aggregates.^33^ The equimolar mixture displayed slightly enhanced low-shear viscosity despite containing half the concentration of 1NO_2_FF, together with stronger shear thinning behaviour. At higher shear rates (>100 s^−1^), the viscosities converged towards those of 1NO_2_FF. Mixing increased low-shear resistance and shear responsiveness, consistent with stronger mesoscale connectivity within the mixed system, although viscosity alone cannot distinguish between co-assembly from other scenarios.^33,34^ Following 420 nm irradiation for 30 min, only minor viscosity changes were observed for all samples. These differences may partially reflect heating during irradiation and cooling after irradiation. Overall, the viscosity measurements primarily highlights the effect of mixing, while irradiation produces only limited changes in bulk flow behaviour under these conditions.

Chiroptical measurements further suggest that the self-assembly in the mixture is directed by 1NO_2_FF. The CD spectra were dominated by far-UV features between 180 and 240 nm, while signals above ∼250 nm remained close to baseline (Fig. 1d and Fig. S13), indicating that the major contributions arise from peptide backbone and short-wavelength aromatic transitions.^35,36^1NO_2_FF displayed a strong positive band below ∼195 nm and an intense negative band centred near 220 nm with a shoulder around 205 nm. This spectral pattern persisted with reduced intensity upon dilution to 5 mM (Fig. S14), indicating retention of the assembled chiral environment.^35,36^ In contrast, 6OMeVF was essentially CD silent across the measured range at both concentrations, reflecting limited aggregation. The mixed system showed the spectral features of 1NO_2_FF (Fig. 1d), suggesting a similar self-assembly environment. Irradiation at 420 nm for 30 min produced only minor spectral changes, with no clear band shifts and only small intensity variations that may also reflect differences in sample loading.^37^

The most pronounced response to irradiation was the visible colour change, which was strongest in the multicomponent system. 6OMeVF remained colourless before and after irradiation, whereas 1NO_2_FF changed from pale yellow to dark yellow-brown (Fig. 1b). The equimolar mixture underwent a pronounced yellow-to-red transition and became slightly translucent following irradiation. UV-vis absorption spectra showed that the individual components were dominated by near-UV absorption with weak visible tails and comparatively small spectral changes after 420 nm irradiation (Fig. 1e). In contrast, the mixed system developed a broad increase in apparent absorbance across the visible region (∼450–800 nm), together with an enhanced long-wavelength tail. This behaviour is consistent with irradiation altering the aggregate-state optical response of the mixed system. Increased turbidity and scattering after irradiation also contribute to elevated baseline. A Job's plot constructed from the absorbance at 550 nm showed increasing intensity with increasing 1NO_2_FF fraction, reaching a maximum near the 1 : 1 composition followed by a broad plateau between 60 and 90 mol% 1NO_2_FF (Fig. 1f). This behaviour suggests that mixed D–A environments favour formation of the visible light absorbing state. Together, these observations indicate that combining the nitro-naphthyl and methoxy-naphthyl dipeptides generates a substantially stronger light response than either component alone under identical conditions. Freeze-dried samples of the yellow and red solutions showed identical NMR spectra (Fig. S15), indicating that irradiation does not cause detectable chemical degradation or covalent modification.

As the irradiated samples became increasingly optically dense and scattering, diffuse reflectance UV-vis spectroscopy was used to further investigate the visible spectral response, as transmission measurements are more strongly affected by turbidity.^38,39^ The single-component systems differed primarily in the extent of their visible tails. 6OMeVF showed a relatively sharp absorption edge with minimal long-wavelength contribution, whereas 1NO_2_FF exhibited a broader decay into the visible region (Fig. 2a). Before irradiation, the equimolar mixture showed an intermediate visible tail. After 420 nm irradiation, however, the mixed sample displayed the most pronounced and persistent long-wavelength tail across the visible region, supporting an irradiation-induced change in its aggregate-state optical response.

**Fig. 2:**
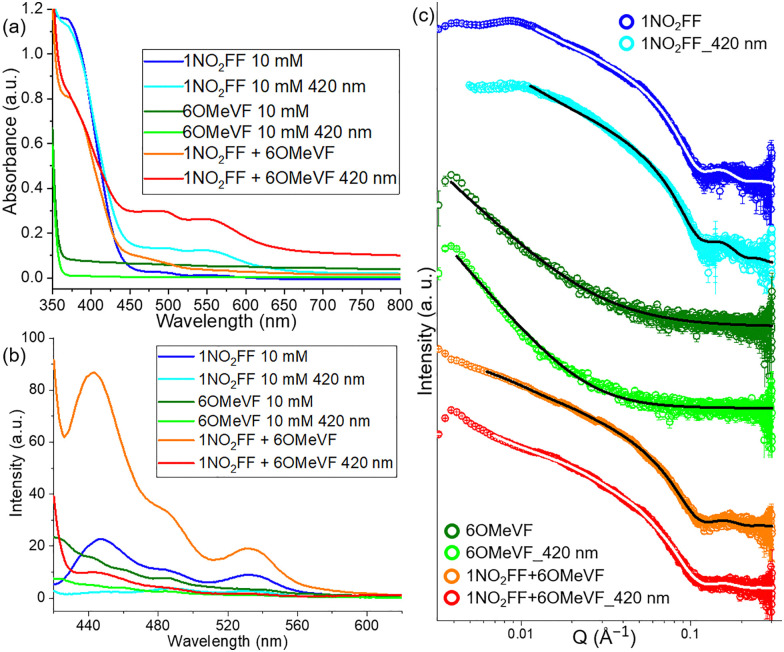
(a) Reflectance UV spectra of 1NO_2_FF (10 mM), 6OMeVF (10 mM), and their equimolar mixture (5 mM each) before and after irradiation at 420 nm (30 min). (b) Photoluminescence emission spectra (λ_ex_ = 400 nm) for the same samples before and after 420 nm irradiation. (c) SAXS of the samples, the data (shown in circles) are stacked on the *y*-axis, and fits are shown in white or black lines. The structure-factor peak for 1NO_2_FF at *q* ≈ 0.01 Å^−1^ was excluded from fitting.^42^ The 2D SAXS pattern of irradiated 1NO_2_FF was anisotropic; therefore, restricted azimuthal integration was performed, losing some data points at low *q* (see SI).

Steady-state emission measurements further support altered electronic interactions upon mixing and substantial suppression of emissive states after irradiation. Emission spectra were collected using excitation at 400 nm to minimise excitation-scattering artefacts and probe visible-emitting states associated with the nitro-naphthyl system (Fig. 2b). Under these conditions, 6OMeVF was essentially non-emissive, with residual intensity arising primarily from scattering. 1NO_2_FF showed weak broad blue emission, whereas the 1 : 1 mixture displayed enhanced and broadened emission despite containing only half the concentration of the nitro-containing component. This suggests that mixing modifies the emissive excited-state environment, consistent with reports of D–A peptide assemblies that generate altered low-energy optical features relative to the individual components.^40^ Following 420 nm irradiation, emission from both 1NO_2_FF and the mixed system was strongly quenched, tracking the visible colour change. This quenching likely reflects a combination of altered excited-state behaviour and increased inner-filter and scattering effects in the darker irradiated samples.^39,41^ The moderate light-induced changes observed for 1NO_2_FF alone are presumably due to interactions with electron-donating species present in the sample medium/atmosphere. To test this, the 1NO_2_FF solution was purged with Ar for 30 min prior to irradiation, after which only a slight colour change to dark yellow was observed (Fig. S16), with reduced changes in UV-vis absorbance and no fluorescence quenching (Fig. S17).

The spectroscopic observations were correlated with nanoscale organisation using SAXS (Fig. 2c and Fig. S18). The scattering profile of 1NO_2_FF was fitted using a Cylinder + Power Law model (Table S1), yielding elongated tubular nanostructures with ∼33 Å radius and ∼436 Å length, with additional low-*q* scattering arising from larger-scale network or interfacial features.^43^6OMeVF was fitted using Power Law model alone, showing that its SAXS profile is dominated by broad low-*q* scattering. The scattering pattern and fitted parameters of the equimolar mixture were comparable to those of 1NO_2_FF (Table S2), with slightly reduced radius (∼31.5 Å) but increased length (∼540 Å). After irradiation at 420 nm, the scattering patterns and fitted models remained the same, but the fitted cylinder length of 1NO_2_FF and the mixture reduced to less than half of the initial value, while the fitted radius remained essentially unchanged. These results suggest that irradiation alters the long-range organisation of the tubular assemblies while largely preserving their cross-sectional dimensions.

Cryo-EM of vitrified solutions revealed clear morphological differences between the single-component systems and the equimolar mixture (Fig. 3 and Fig. S19–S24).^15,44^1NO_2_FF formed a relatively uniform population of long nanotube-like assemblies (Fig. S19), with diameters consistent with the SAXS-derived values (∼6.5 nm). 6OMeVF was morphologically heterogeneous, containing thin fibrils together with thicker fibre- or tape-like aggregates and occasional twisted or helical features (Fig. S21). The heterogeneity likely made the average SAXS profile less defined, so the data fitted in a Power Law model. The 1 : 1 mixture displayed uniform nanotube-like structures resembling those observed for 1NO_2_FF, with no obvious second population corresponding to the assemblies formed by 6OMeVF alone (Fig. S23). These observations support the formation of a mixed assembled state rather than simple coexistence of two independent morphologies.

**Fig. 3:**
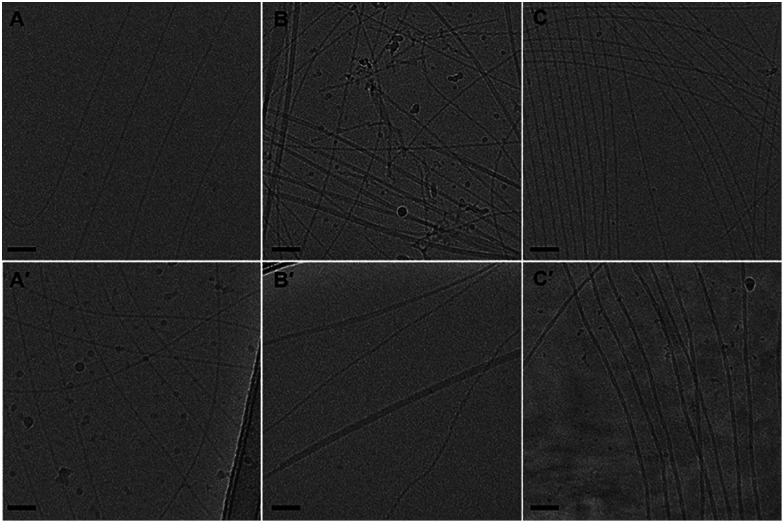
Cryo-EM images of vitrified solutions of (A) 1NO_2_FF, (B) **6OMeVF**, and (C) equimolar 1NO_2_FF + 6OMeVF (5 mM each). A′–C′ correspond samples after irradiation under a solar simulator for 30 min. Additional figures are provided in SI (Fig. S19–S24). Scale bars = 50 nm.

Following irradiation under a solar simulator for 30 min, both 1NO_2_FF and 6OMeVF retained their dominant morphologies with no major restructuring apparent in Cryo-EM (Fig. S20 and S22). The irradiated multicomponent sample also remained dominated by tubular assemblies, although some nanotubes appeared less regular than in the untreated sample, showing local distortions and occasional flattened or twisted features (Fig. S24). Overall, the Cryo-EM data indicate that irradiation does not destroy the tubular assemblies, but induces more subtle changes in aggregate organisation within the mixed system. This interpretation is consistent with the SAXS analysis, where the fitted radii remain largely unchanged whereas the fitted cylinder lengths decrease substantially after irradiation. These observations support light-induced modulation within the multicomponent assembled state rather than a complete change in aggregate morphology.

In summary, complementary electron-rich and electron-deficient groups can control multicomponent peptide assembly, while light induces reversible structural modulation at the nanoscale, providing a simple route to responsive supramolecular materials.

## Conflicts of interest

There are no conflicts to declare.

## Supplementary Material

CC-062-D6CC03505A-s001

## Data Availability

The supporting data have been provided as part of the supplementary information (SI). Supplementary information: synthesis and characterisation of the gelators, further experimental details, additional viscosity, CD data, SAXS tables, and additional Cryo-EM images. See DOI: https://doi.org/10.1039/d6cc03505a.
