# Supplementary material for: Light-triggered modulation in donor–acceptor dipeptide assemblies
Source: Chem Commun (Camb). 2026 Jul 13;62(61):15325–9. doi: 10.1039/d6cc03505a (PMC13382782; doi:10.1039/d6cc03505a)
Supplement: CC-062-D6CC03505A-s001 [file CC-062-D6CC03505A-s001.pdf]

Supplementary Information (SI) for ChemComm.
This journal is © The Royal Society of Chemistry 2026

Supplementary information

Light-Triggered Modulation in Donor-Acceptor Dipeptide Assemblies

*Dipankar Ghosh,[a] Ravi R. Sonani,[b] Chengcheng Zhao,[a] Simona Bianco,[c] Edward H. Egelman,[b] Duncan H. Gregory [a] and Dave J. Adams * [a]*

Experimental

Materials and methods

Reagents and solvents were purchased from commercial suppliers (Sigma-Aldrich or Fluorochem Ltd) and used as received. Deionised water was used for all aqueous preparations. The peptide conjugates FF-OMe and VF-OMe (TFA salts) and the capping acids 1NO_2-NapOCH_2COOH (for **1NO_2FF**) and 6OMe-NapOCH_2COOH (for **6OMeVF**) were synthesised according to reported procedures.[1, 2] Synthetic details for the gelators used here are provided below.

General synthesis of 1NO_2-2NapFF (1NO_2FF) and 6OMe-2NapVF (6OMeVF)

The required *N*-terminal naphthalene capping acid (5.0 mmol, 1.0 equiv.) was dissolved in $CHCl_3$ (70 mL) and cooled to 0 to 5 °C. *N*-Methylmorpholine (6.0 mmol, 1.2 equiv.) was added, followed by isobutyl chloroformate (6.0 mmol, 1.2 equiv.), and the mixture was stirred for 1 h at 0 to 5 °C to form the mixed anhydride. In parallel, the corresponding ester-protected dipeptide (FF-OMe for **1NO_2FF** or VF-OMe for **6OMeVF**; as the TFA salt; 5.0 mmol, 1.0 equiv.) was suspended in $CHCl_3$ (50 mL) at 0 to 5 °C and neutralised with *N*-methylmorpholine (7.0 mmol, 1.4 equiv.) to generate the free amine. This peptide solution was added to the mixed anhydride, and the reaction was allowed to warm to room temperature and stirred overnight. The mixture was diluted with $CHCl_3$ and washed successively with 1 M HCl, water, and brine. The organic layer was dried over $MgSO_4$, filtered, and concentrated to give the crude coupled ester, which was used directly in the next step.

For ester hydrolysis, the crude product (4.0 mmol, 1.0 equiv.) was dissolved in THF (20 mL) and treated with aqueous LiOH (1.0 M, 20 mL, 5 molar equiv.). The reaction was stirred at room temperature

overnight (typically becoming homogeneous as hydrolysis progressed), then poured into 0.5 M HCl (250 mL) and stirred for 60 to 90 min to ensure complete protonation and precipitation. The resulting solid was collected by filtration, washed thoroughly with water, and dried under vacuum to afford the corresponding N-capped peptide gelators **1NO$_2$FF** or **6OMeVF**.

Characterization of the peptides

(2-((1-Nitronaphthalen-2-yl)oxy)acetyl)-L-phenylalanyl-L-phenylalanine (**1NO$_2$FF**)

^{1}H NMR (400 MHz, DMSO-d$_6$) δ 12.81 (s, 1H), 8.48 (d, *J* = 7.9 Hz, 1H), 8.18 (d, *J* = 8.7 Hz, 1H), 8.05 (dd, *J* = 8.8, 3.6 Hz, 2H), 7.69 (ddd, *J* = 8.3, 6.8, 1.2 Hz, 1H), 7.61 – 7.52 (m, 2H), 7.29 – 7.13 (m, 11H), 4.76 (d, *J* = 1.7 Hz, 2H), 4.69 – 4.61 (m, 1H), 4.48 (td, *J* = 8.4, 5.3 Hz, 1H), 3.13 – 3.00 (m, 2H), 2.93 (dd, *J* = 13.9, 8.9 Hz, 1H), 2.73 (dd, *J* = 13.8, 10.0 Hz, 1H).

^{13}C NMR (101 MHz, DMSO-d$_6$) δ 172.68, 170.68, 166.33, 147.16, 137.40, 137.37, 134.89, 132.35, 129.50, 129.20, 129.10, 128.34, 128.17, 128.02, 127.87, 126.42, 126.29, 125.42, 124.56, 119.59, 114.52, 67.44, 53.50, 53.20, 37.77, 36.66.

HRMS [M-H]- calculated for $[C_{30}H_{26}N_3O_7]^-$: 540.1776, found 540.1786.

(2-((6-Methoxynaphthalen-2-yl)oxy)acetyl)-L-valyl-L-phenylalanine (**6OMeVF**)

^{1}H NMR (400 MHz, DMSO-d$_6$) δ 12.69 (s, 1H), 8.40 (d, *J* = 7.7 Hz, 1H), 7.82 (d, *J* = 9.1 Hz, 1H), 7.75 (d, *J* = 8.9 Hz, 1H), 7.66 (d, *J* = 9.0 Hz, 1H), 7.29 – 7.11 (m, 9H), 4.69 – 4.58 (m, 2H), 4.45 (ddd, *J* = 9.2, 7.7, 5.3 Hz, 1H), 4.31 (dd, *J* = 9.2, 6.4 Hz, 1H), 3.84 (s, 3H), 3.05 (dd, *J* = 14.0, 5.3 Hz, 1H), 2.88 (dd, *J* = 14.0, 9.2 Hz, 1H), 1.99 (h, *J* = 6.7 Hz, 1H), 0.85 – 0.73 (m, 6H).

^{13}C NMR (101 MHz, DMSO-d$_6$) δ 172.70, 170.60, 167.30, 155.86, 153.93, 137.45, 129.73, 129.13, 129.02, 128.20, 128.12, 128.07, 126.37, 118.87, 118.62, 107.70, 106.14, 66.81, 56.76, 55.09, 53.38, 36.58, 30.95, 19.12, 17.70.

HRMS [M-H]- calculated for $[C_{27}H_{29}N_2O_6]^-$: 477.2031, found 477.2036.

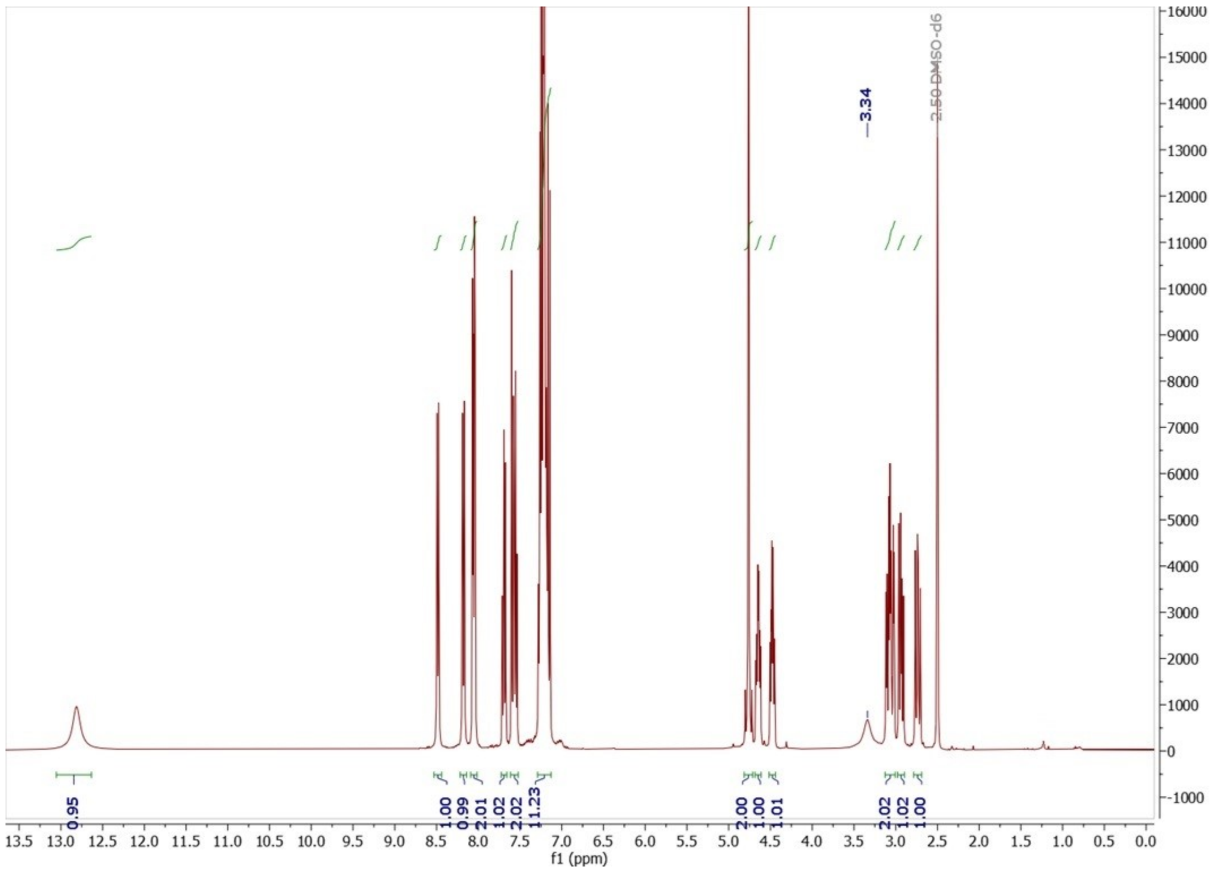

Fig. S1. ^{1}H-NMR of **1NO$_2$FF**.

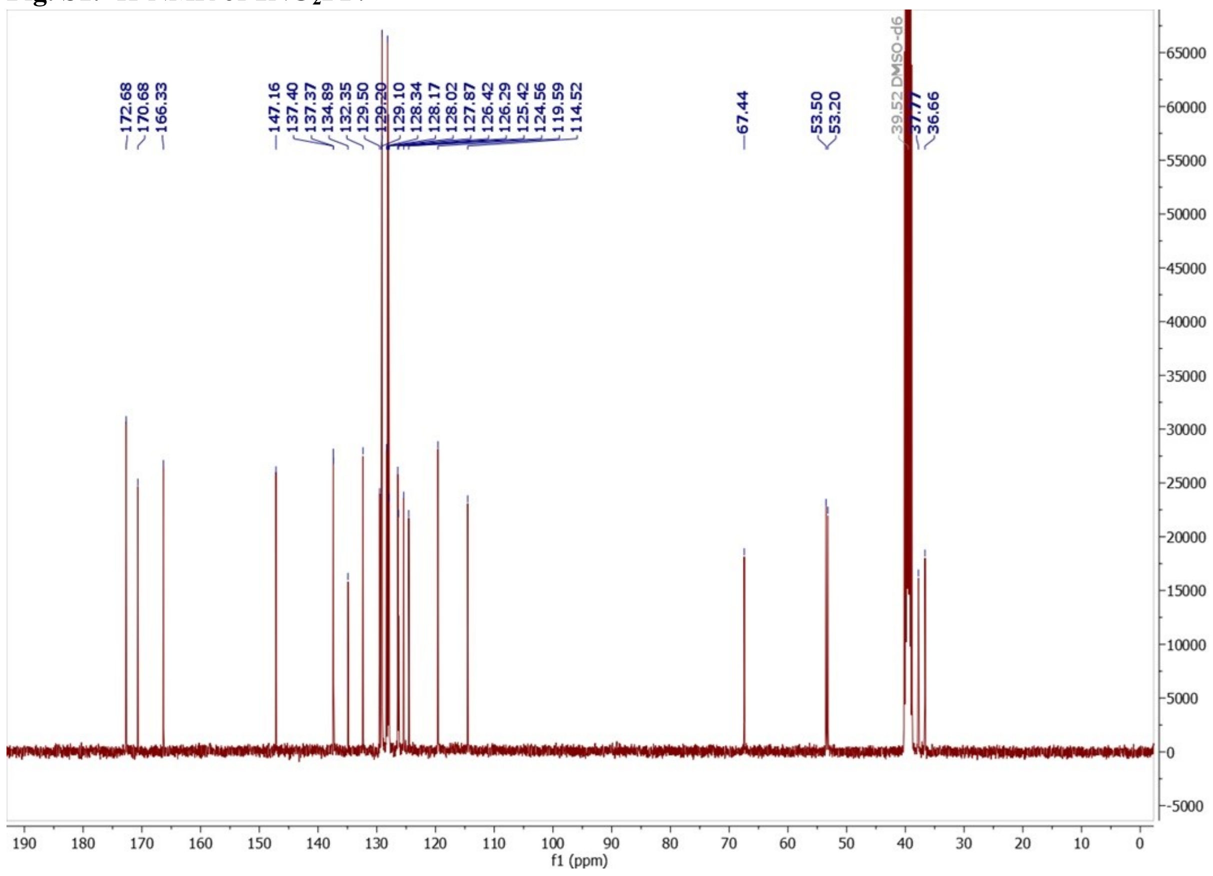

Fig. S2. ^{13}C-NMR of **1NO$_2$FF**.

Compound Details

Cpd. 1: C30 H27 N3 O7

Name	Formula	Mass (Tgt)	Mass	Diff (Tgt, ppm)	Score (Tgt)
	C30 H27 N3 O7	541.184900	541.185787	1.64	98.91

Compound Spectra (overlaid)

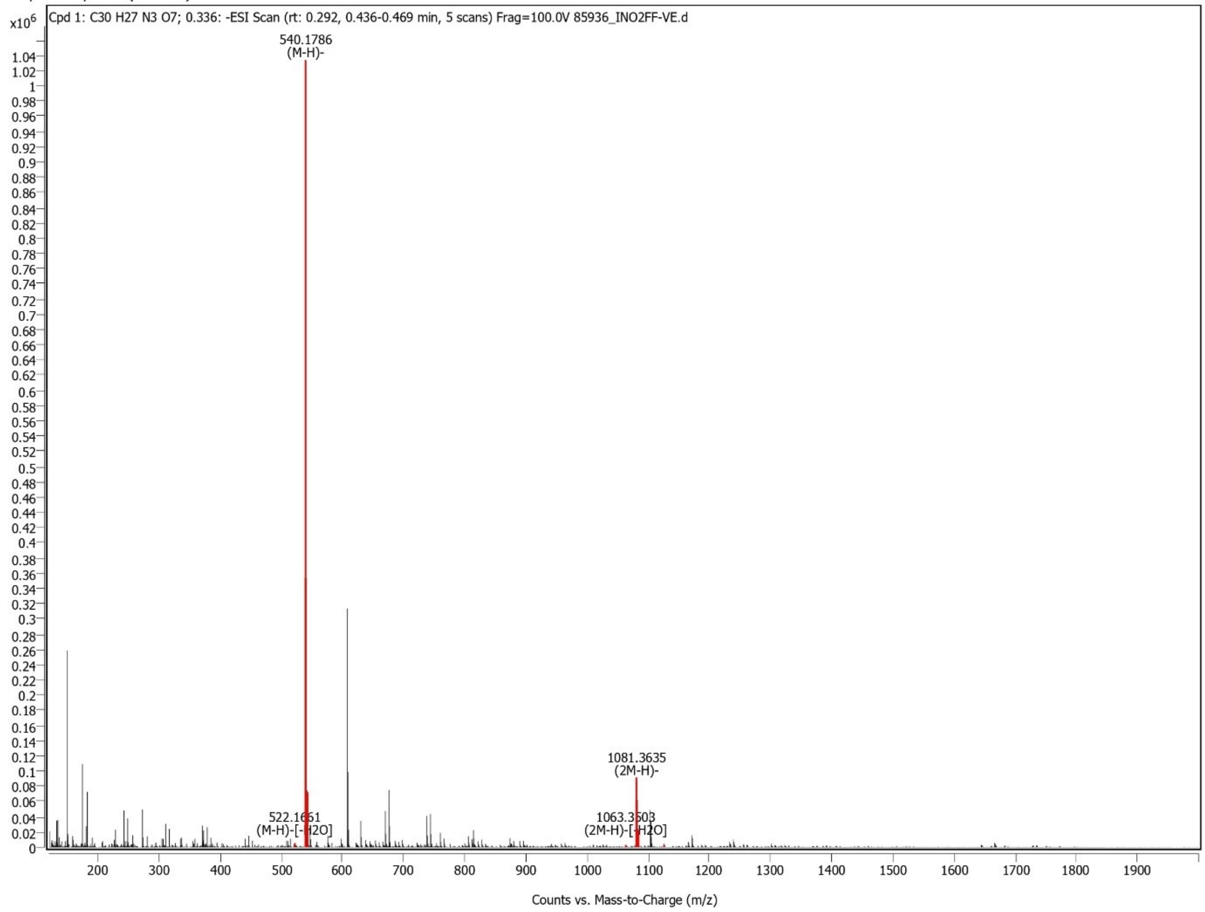

Fig. S3. HRMS of **1NO$_2$FF**.

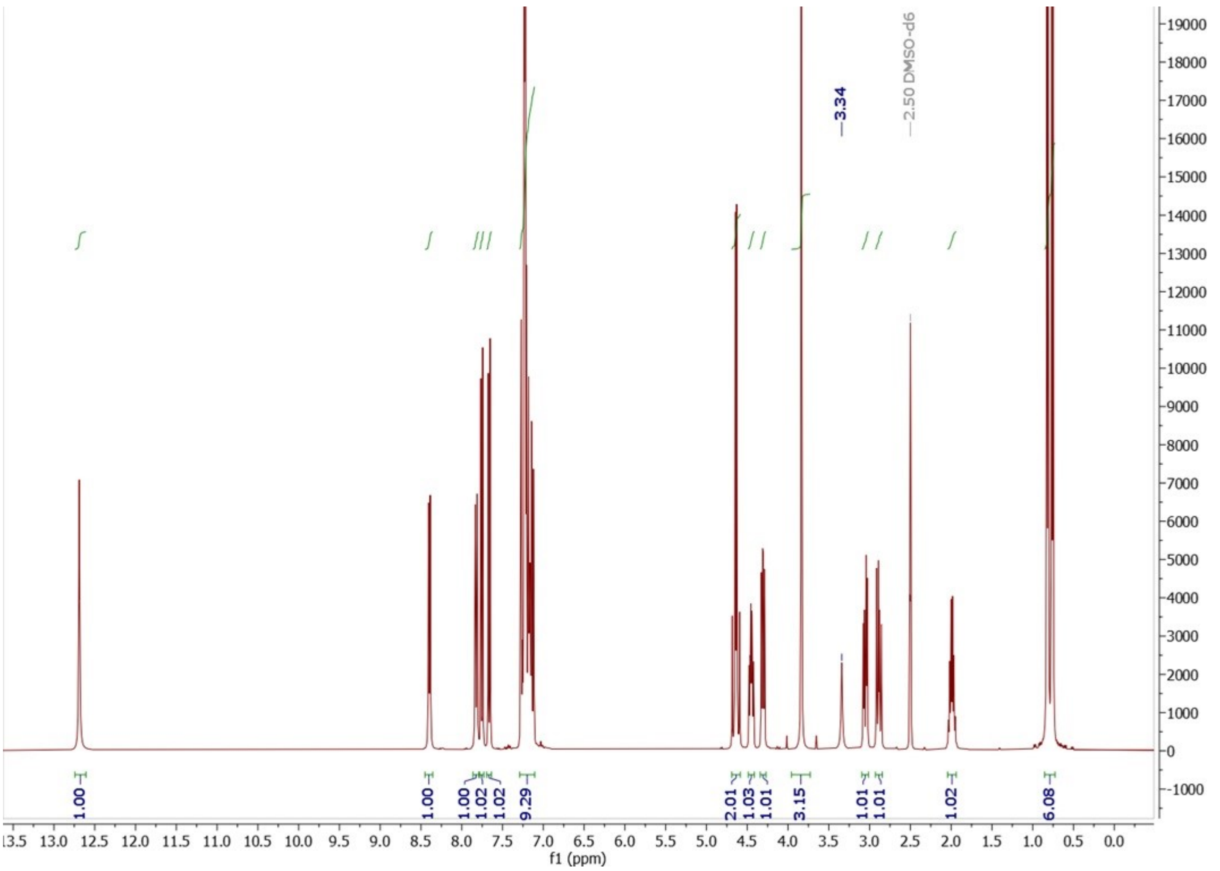

Fig. S4. ^{1}H-NMR of **6OMeVF**.

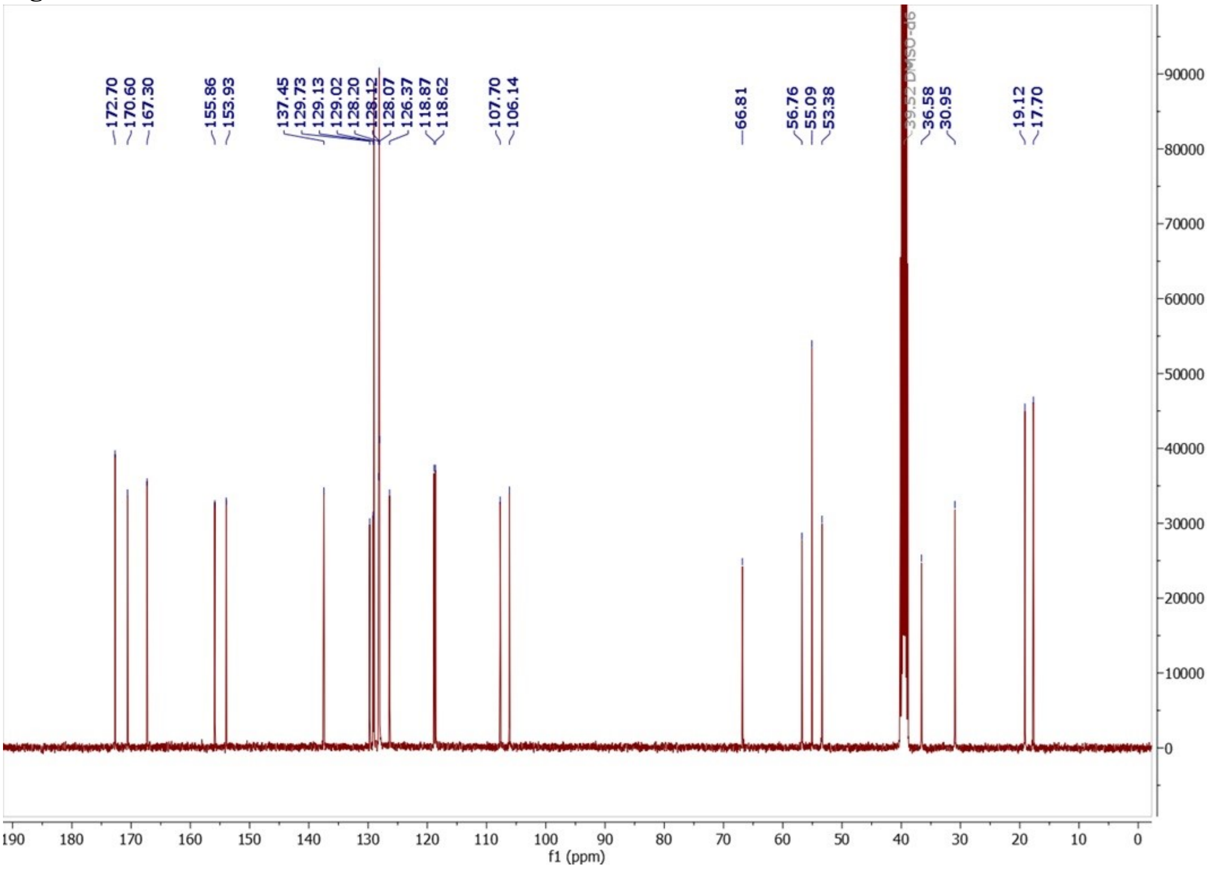

Fig. S5. ^{13}C-NMR of **6OMeVF**.

Compound Details

Cpd. 1: C27 H30 N2 O6

Name	Formula	Mass (Tgt)	Mass	Diff (Tgt, ppm)	Score (Tgt)
	C27 H30 N2 O6	478.210387	478.210765	0.79	99.40

Compound Spectra (overlaid)

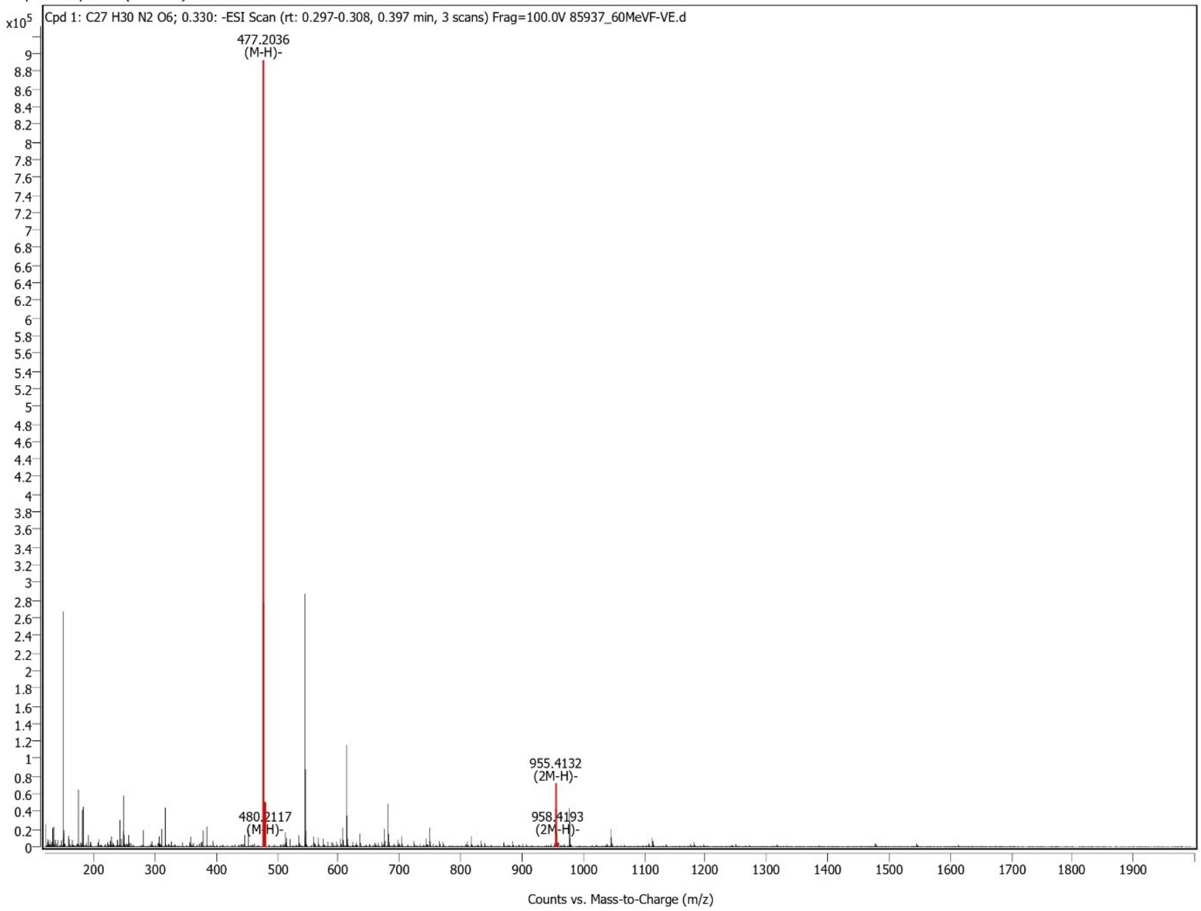

Fig. S6. HRMS of **6OMeVF**.

Preparation of peptide solutions

Single-component stock solutions (10 mM) were prepared by weighing the gelator (50 µmol; ~27 mg for **1NO$_2$FF** or ~24 mg for **6OMeVF**) into a 7 mL glass vial (outer diameter 17 mm), then adding deionised water (4.5 mL) and freshly prepared NaOH (0.1 M, 500 µL; 1.0 equiv.). The mixtures were stirred overnight at room temperature at 500 rpm using a 3 × 13 mm cylindrical stirrer bar. The multicomponent sample was prepared by mixing 25 µmol of each gelator (as solids) into the same type of vial and preparing the solution in an identical manner (giving 5 mM of each component).

Light irradiation conditions

Irradiation experiments were performed using either a solar simulator (SunLite, ABET Technologies) or high-power LED sources (Luxeon C LED module, 1-LED). Samples were placed inside a 7 mL glass vial (OD 17 mm), sealed with a plastic screw cap and irradiated for 30 min at ambient temperature with stirring on an IKA Topolino stirring plate (ca. 500 rpm) using a 3 × 13 mm cylindrical stirrer bar. Samples were positioned at ~5 cm from the LED or the solar simulator. For wavelength screening, LEDs centred at 420, 455, 470, 505, and 565 nm were used. After optimisation, a 420 nm LED was selected for subsequent experiments. Unless noted otherwise, samples were irradiated at 420 nm for 30 min.

Viscosity measurements

Viscosity measurements were performed on an Anton Paar Physica MCR 101 rheometer using a 50 mm diameter, 1 ° cone-plate geometry (CP50) at 25 °C. The instrument-defined truncation gap was 0.101 mm. Approximately 0.7 mL of sample was loaded onto the plate with a Pasteur pipette to minimise pre-shear. Measurements were performed in triplicate, and results are reported as mean values with standard deviations. Viscosity profiles are reported for **1NO$_2$FF** (10 mM and 5 mM), **6OMeVF** (10 mM and 5 mM), and the equimolar mixture (5 mM each), before and after 420 nm irradiation (30 min).

Circular dichroism (CD) spectroscopy

CD spectra were recorded on a Chirascan CD spectrometer (Applied Photophysics) at 25 °C from 180 - 400 nm using a step size of 1 nm and a bandwidth of 1 nm. Samples (**1NO$_2$FF**, **6OMeVF**, and the equimolar mixture at the concentrations stated above) were measured before and after irradiation using demountable quartz cells with a 0.01 mm path length. A drop of sample was placed on the spacer window, spread evenly, and the cell was sealed with the flat quartz window. Any excess liquid at the edges was wiped off before loading. Spectra were collected in triplicate and averaged. Deionised water was used as the background and was subtracted from all measurements.

UV-vis absorption spectroscopy

UV-vis absorption spectra were recorded on a Cary 60 UV-vis spectrophotometer (Agilent Technologies) using quartz cuvettes with 5 mm path length. Spectra were collected for **1NO$_2$FF** (10 mM), **6OMeVF** (10 mM), and the equimolar mixture (5 mM each), before and after irradiation, over

350 - 800 nm at 300 nm min^{-1} scan rate. Deionised water was used as the reference and was subtracted from all measurements.

Reflectance UV-vis spectroscopy

Diffuse reflectance UV-vis measurements were performed on the single- and multicomponent samples before and after irradiation (420 nm, 30 min). The spectra were recorded on a Shimadzu UV-2600 UV-Vis spectrophotometer with a reflectance attachment at room temperature. The samples were diluted 5-fold with deionised water (20% of the sample + 80% DI water) and measured in a quartz cuvette with an optical path length of 1 cm. Deionised water was used as the blank reference for baseline correction, and spectra were collected over the wavelength range of 1300-190 nm.

Photoluminescence spectroscopy

Photoluminescence spectra were collected on a Cary Eclipse fluorescence spectrophotometer (Agilent Technologies) using four-side transparent quartz cuvettes (10 mm path length). Samples were excited at 400 nm and emission spectra were recorded from 415 to 900 nm at 100 nm min^{-1} scan rate, with excitation and emission slit widths of 20 nm.

Small-angle X-ray scattering (SAXS)

SAXS measurements were carried out on the CoSAXS beamline at MAX IV laboratory (Fotongatan 2, 224 84 Lund, Sweden).[3] The beamline was operated at an energy of 12.4 keV with a camera length of 3.434 m, providing a Q range of 0.00325 to 0.302 Å^{-1}. Samples were prepared as described above and introduced into capillaries (1.5 mm ID) using a syringe fitted with a 21G needle. For each sample, 20 frames were collected along the capillary length with exposure times of 0.5 s. The 2D scattering images were reduced in DAWN Science (v2.40)[4] to generate I vs Q profiles. The water background was subtracted and the 1D scattering data were fitted in SasView (v5.0.6).[5] For the irradiated **1NO$_2$FF** sample, the 2D SAXS pattern showed anisotropic scattering; therefore, sector integration over a reduced azimuthal range of $\phi = -50°$ to 60° was performed. The water background was integrated over the same azimuthal sector before subtraction from the sample data.

Cryogenic Electron Microscopy (Cryo-EM)

Samples, for cryo-EM, were prepared as 10 mM solutions of **1NO$_2$FF** and **6OMeVF**, and as an equimolar mixture (5 mM each), as described above. Samples were stirred under a solar simulator for 30 min and imaged before and after irradiation under cryogenic conditions. Samples were vitrified on lacey carbon grids using a Vitrobot Mark IV system (Thermo Fisher Scientific). Prior to sample application, grids were glow discharged in a GloQube instrument (Quorum Technologies) to render the carbon surface hydrophilic. Approximately 3 µL of sample was applied to the freshly glow-discharged grids, and excess liquid was removed using Whatman filter paper to generate a thin aqueous layer of the sample. Grids were subsequently plunge-frozen in liquid ethane.

Cryo-electron microscopy data were collected using a 200 keV Glacios microscope (Thermo Fisher Scientific) equipped with a cryo-autoloader, an XFEG electron source, and a Falcon4 direct electron detector. Imaging was performed at the University of Virginia Molecular Electron Microscopy Core facility.

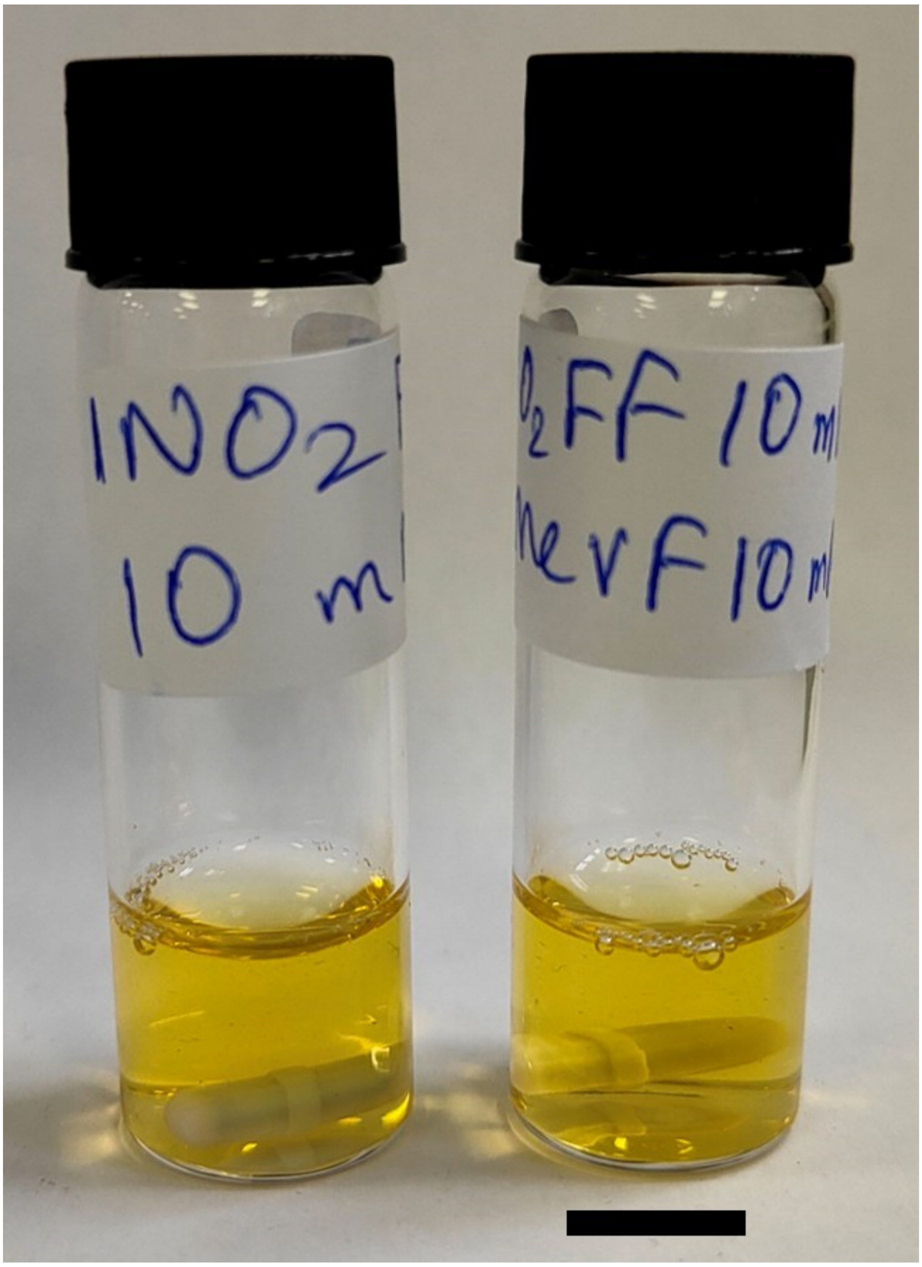

Fig. S7. Photograph of (left) 10 mM **1NO$_2$FF** and (right) equimolar mixture of **1NO$_2$FF** and **6OMeVF**, with each compound present at 10 mM. The double concentration mixture was used to visually compare samples with the same **1NO$_2$FF** concentration, confirming that adding **6OMeVF** does not noticeably change the colour under ambient conditions. Scale bar = 1 cm.

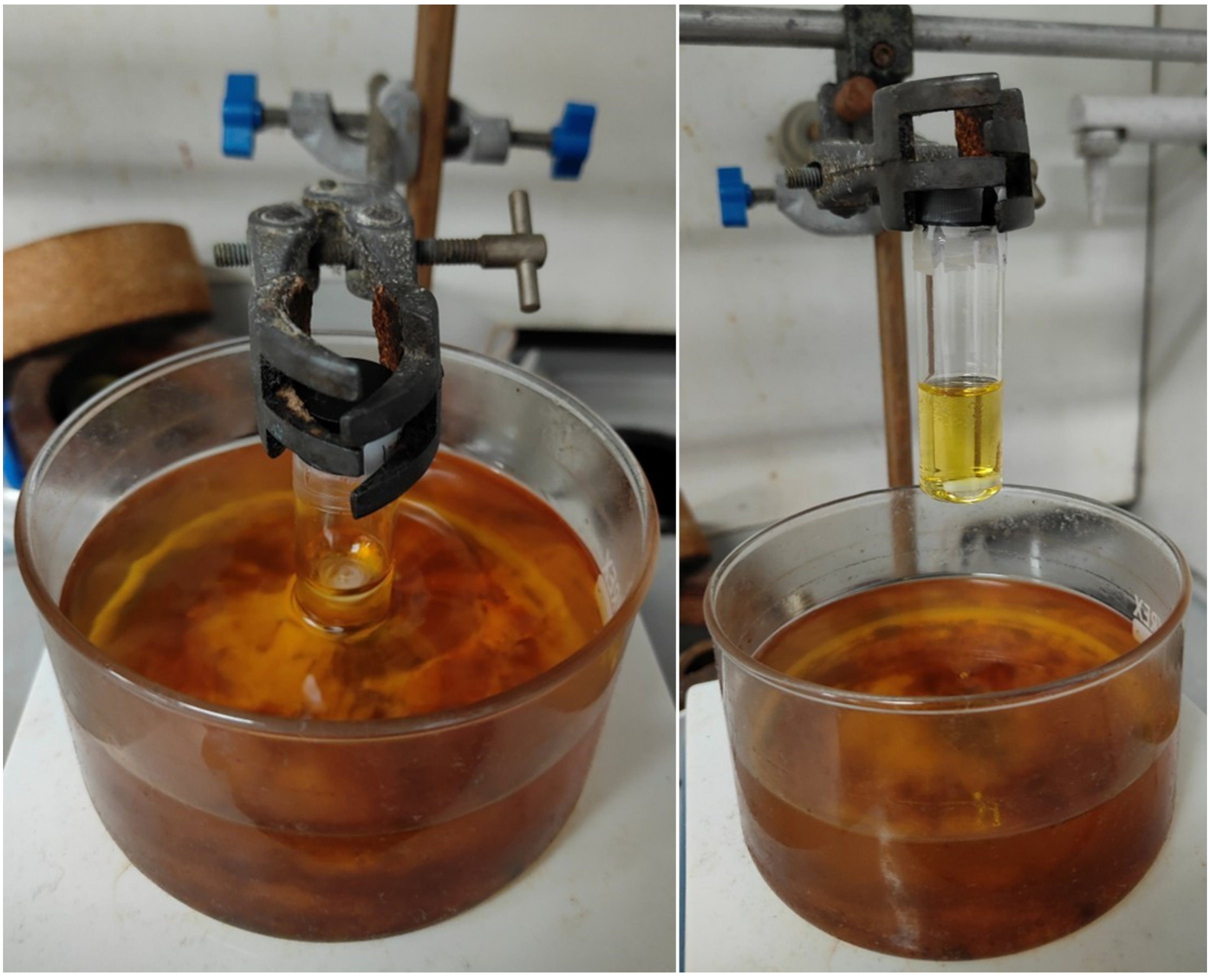

Fig. S8: Experimental set up to test the effect of temperature (75 °C for 2 h) on the **1NO$_2$FF+6OMeVF** solution (5 mM each), showing no change upon heat-cooling.

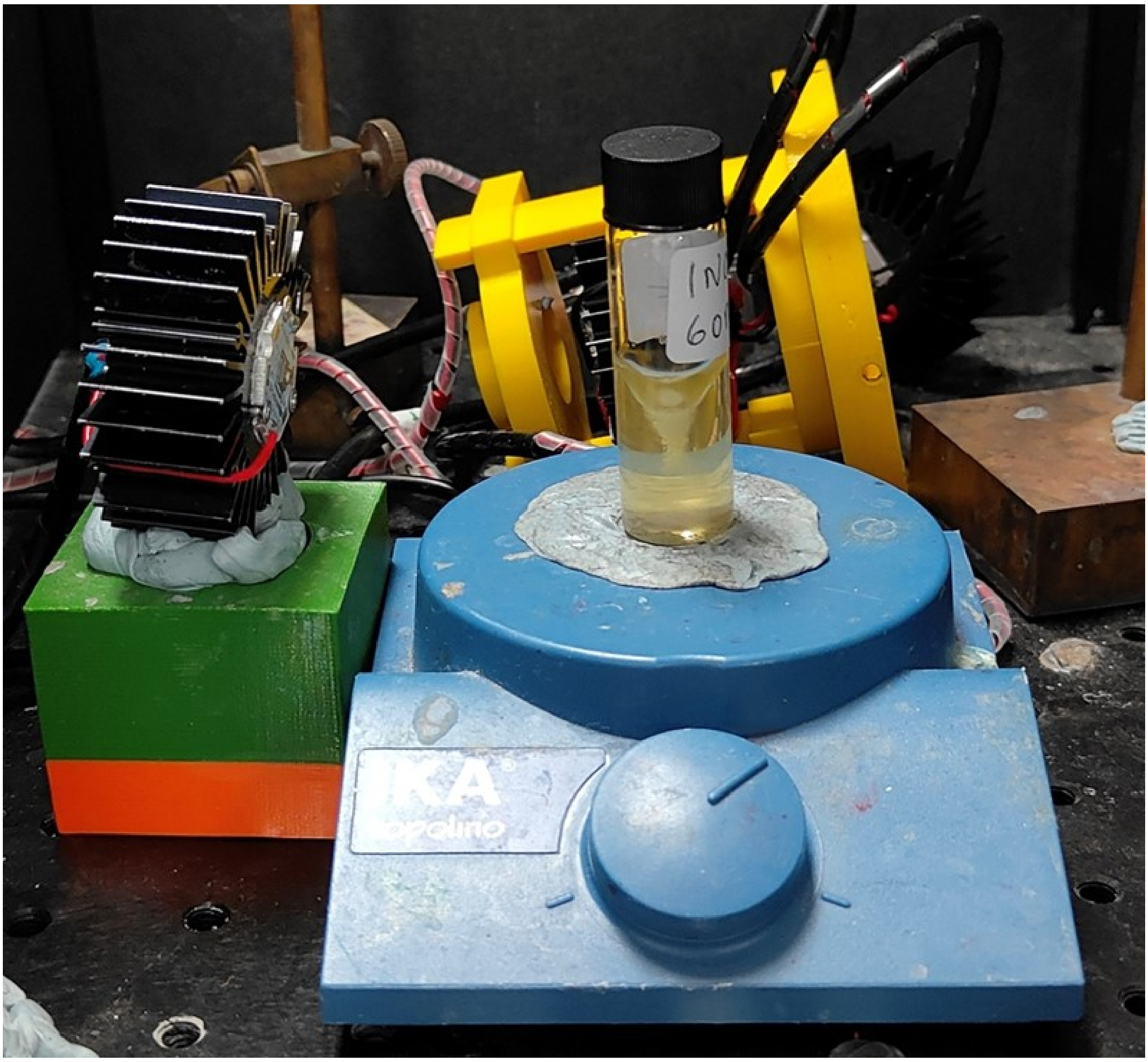

Fig. S9. Experimental set up for the LED irradiation.

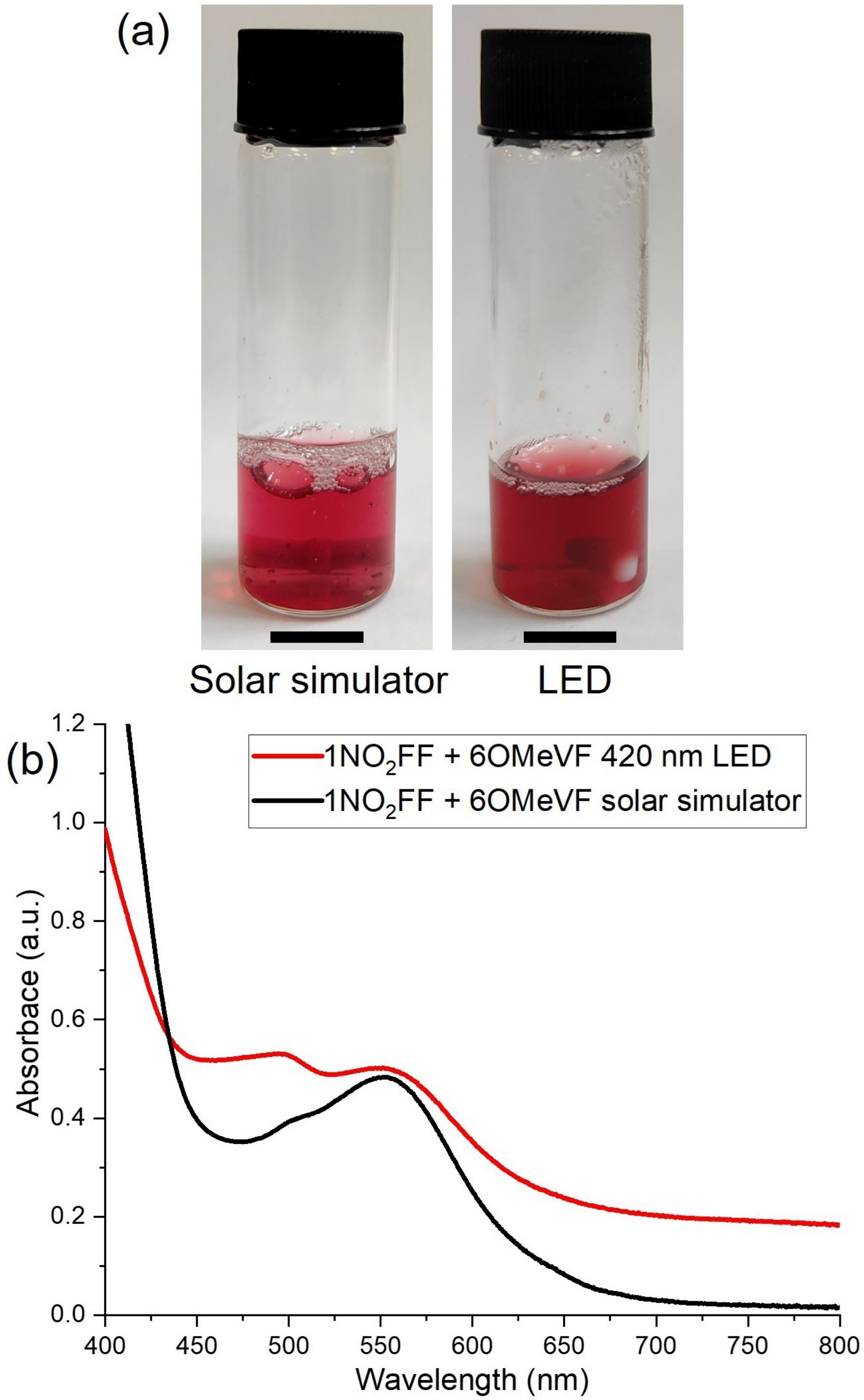

Fig. S10. Comparison of the solar simulator and 420 nm LED irradiation. (a) Photographs of the solutions containing 1:1 mixture of **1NO$_2$FF** and **6OMeVF** after 30 min irradiation using the solar simulator (left) and 420 nm LED (right). Scale bar = 1 cm. (b) UV-vis absorption spectra of the corresponding solutions recorded using a 5 mm path length cuvette.

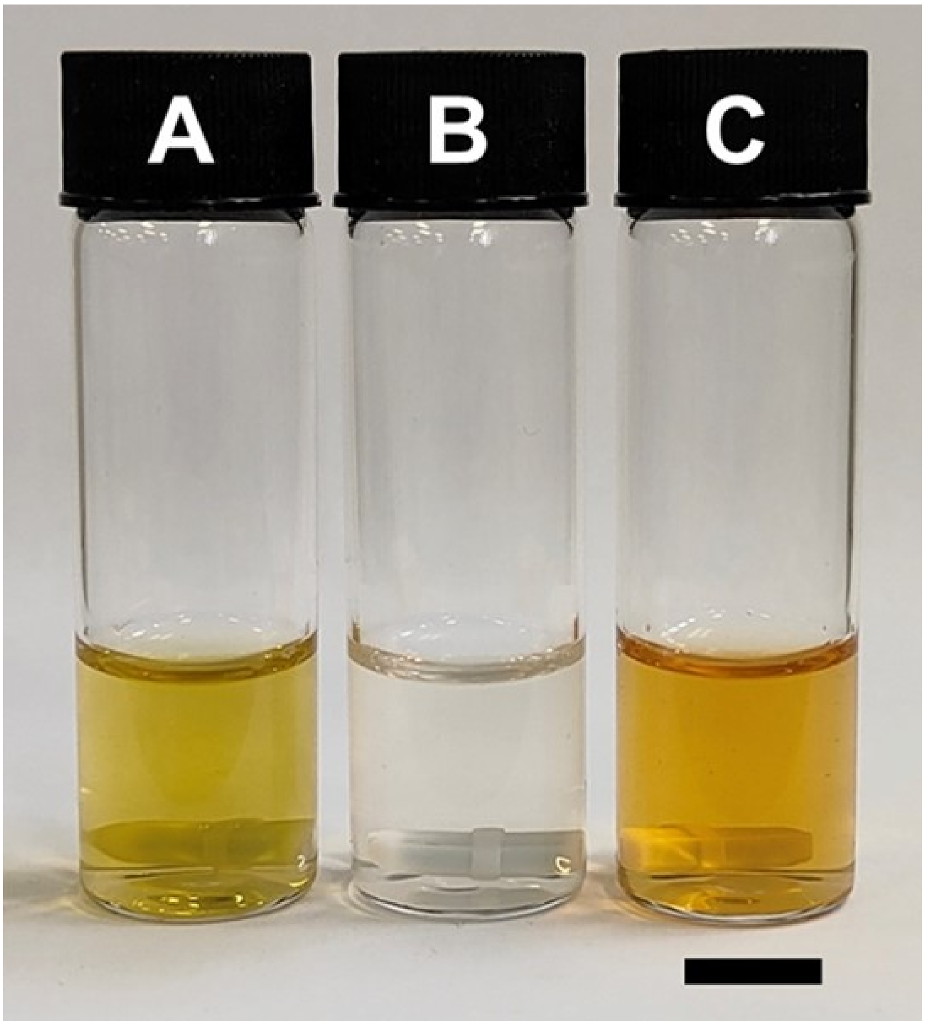

Fig. S11. Photographs of solutions after 14 d of recovery in the dark: [A] **1NO$_2$FF** (10 mM); [B] **6OMeVF** (10 mM); [C] equimolar mixture (5 mM each). Scale bar = 1 cm.

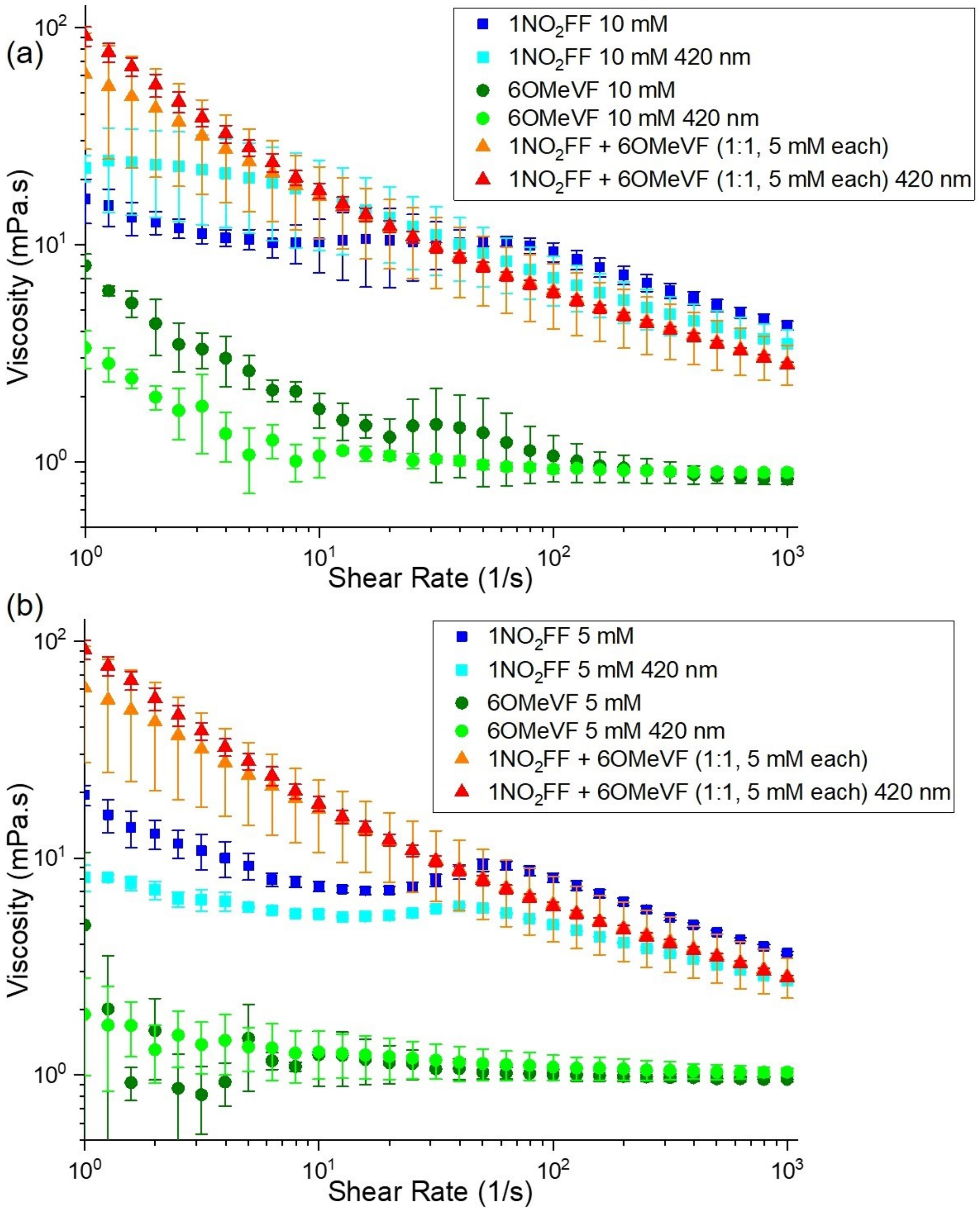

Fig. S12. Viscosity comparison of the single- and multicomponent systems before and after 420 nm irradiation. (a) Single-component samples are 10 mM, while the mixture contains 5 mM of each component (10 mM total). (b) Single-component samples are 5 mM, matching the concentration of each component in the mixture. The multicomponent graphs (orange and red) in both (a) and (b) were plotted from identical data for comparison.

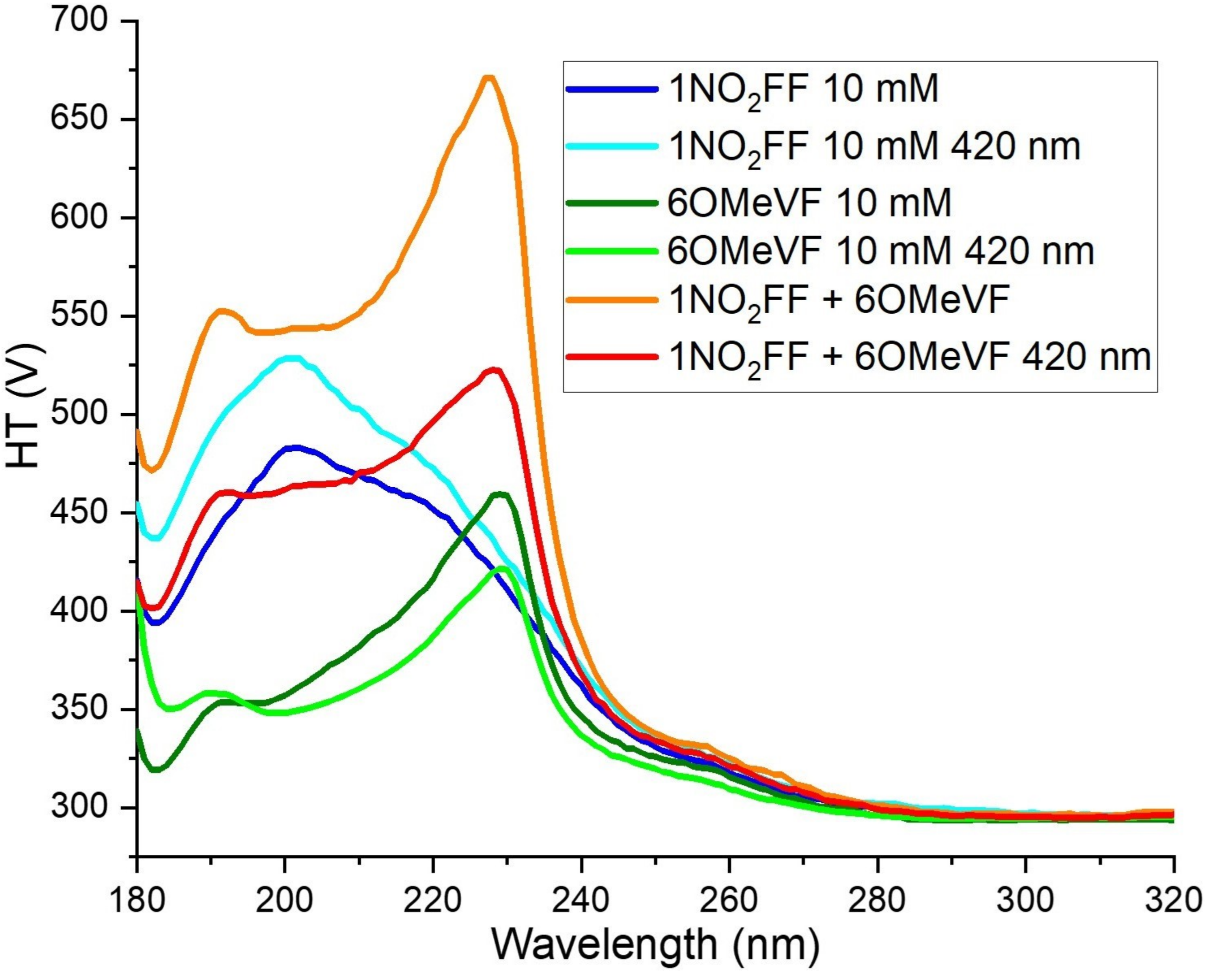

Fig. S13. HT profiles for the CD spectra shown in Fig. 1d in the main text.

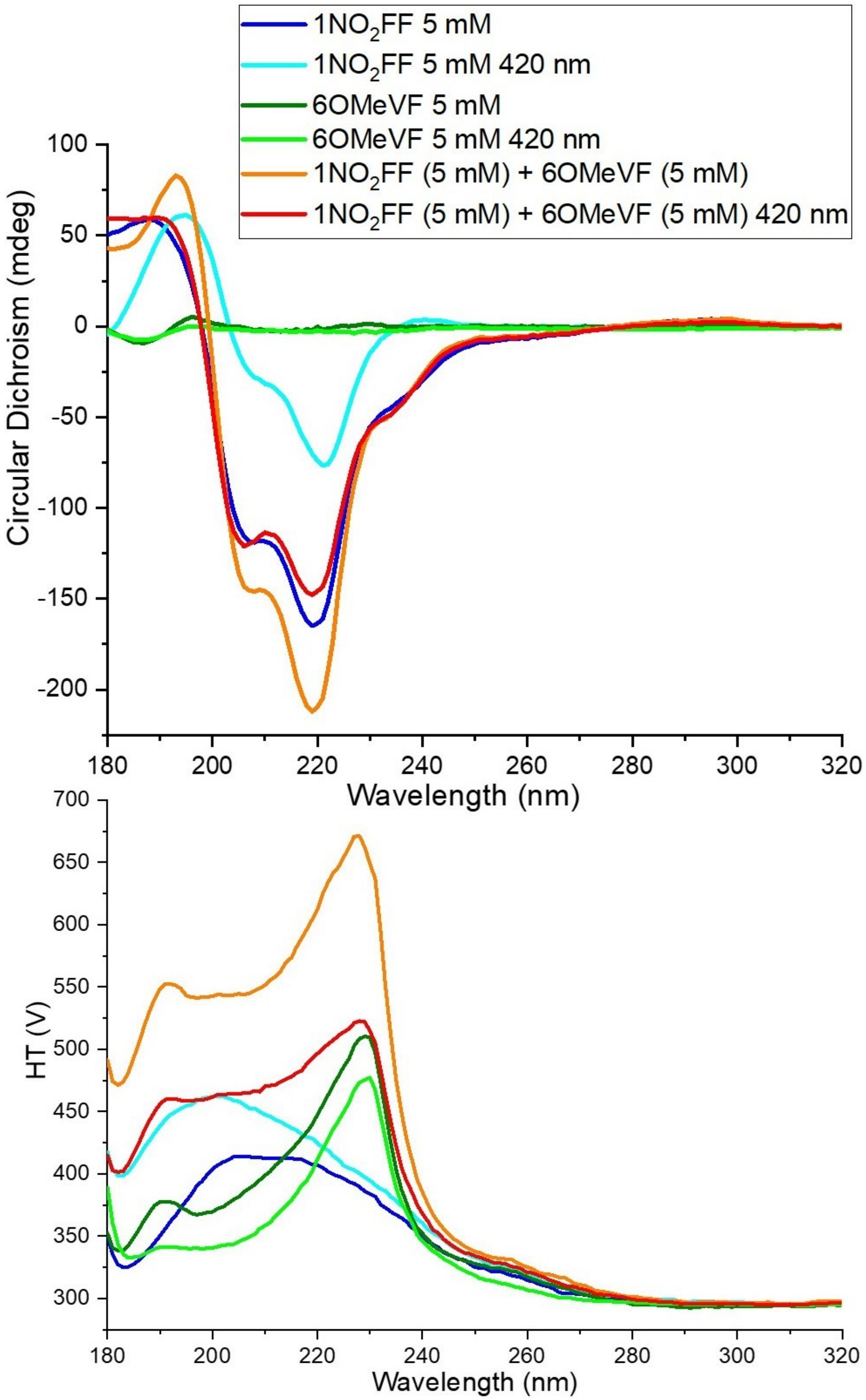

Fig. S14. (top) CD spectra of the single component (**1NO$_2$FF** and **6OMeVF**) solutions at 5 mM concentrations and the mixture where both individual components are at 5 mM (10 mM in total); before and after 420 nm irradiation. (bottom) HT profiles for the same samples.

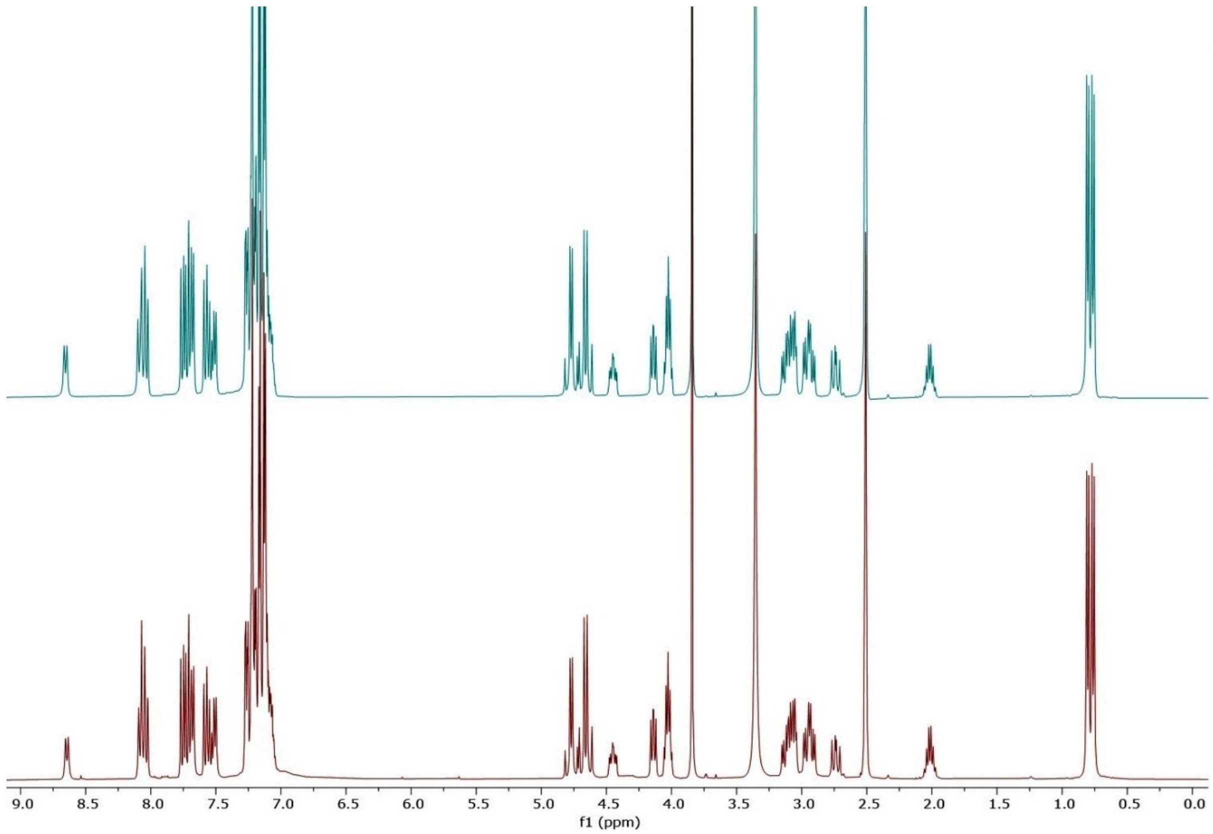

Fig. S15. ^{1}H-NMR of the freeze-dried 1:1 mixture of **$1NO_2FF$** and **6OMeVF**, before irradiation (top) and after 420 nm irradiation for 30 min (bottom).

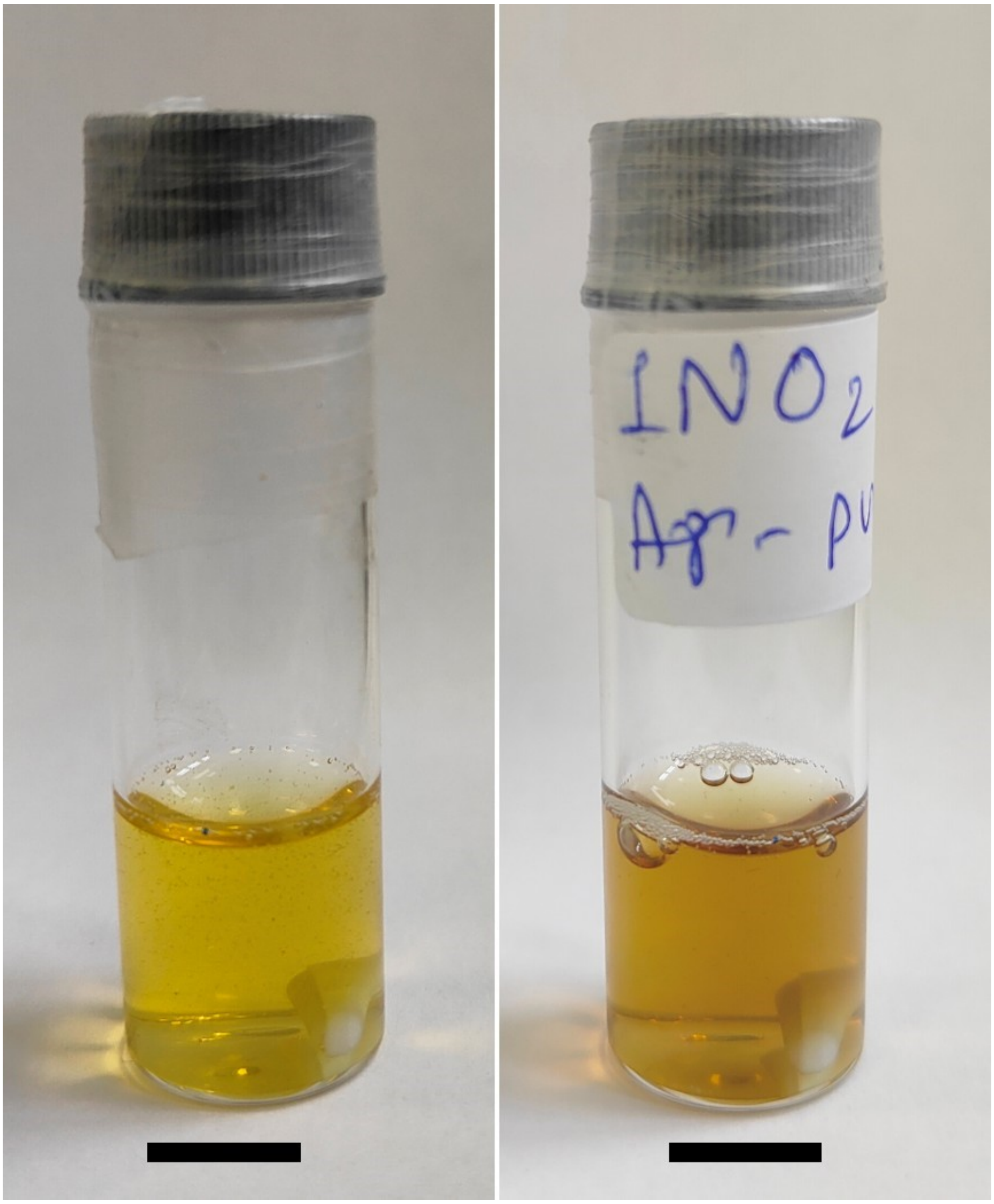

Fig. S16. Photographs of **$1NO_2FF$** purged with Ar for 30 min (left) and the sample after irradiation for 30 min under a 420 nm LED (right). Scale bars = 1 cm.

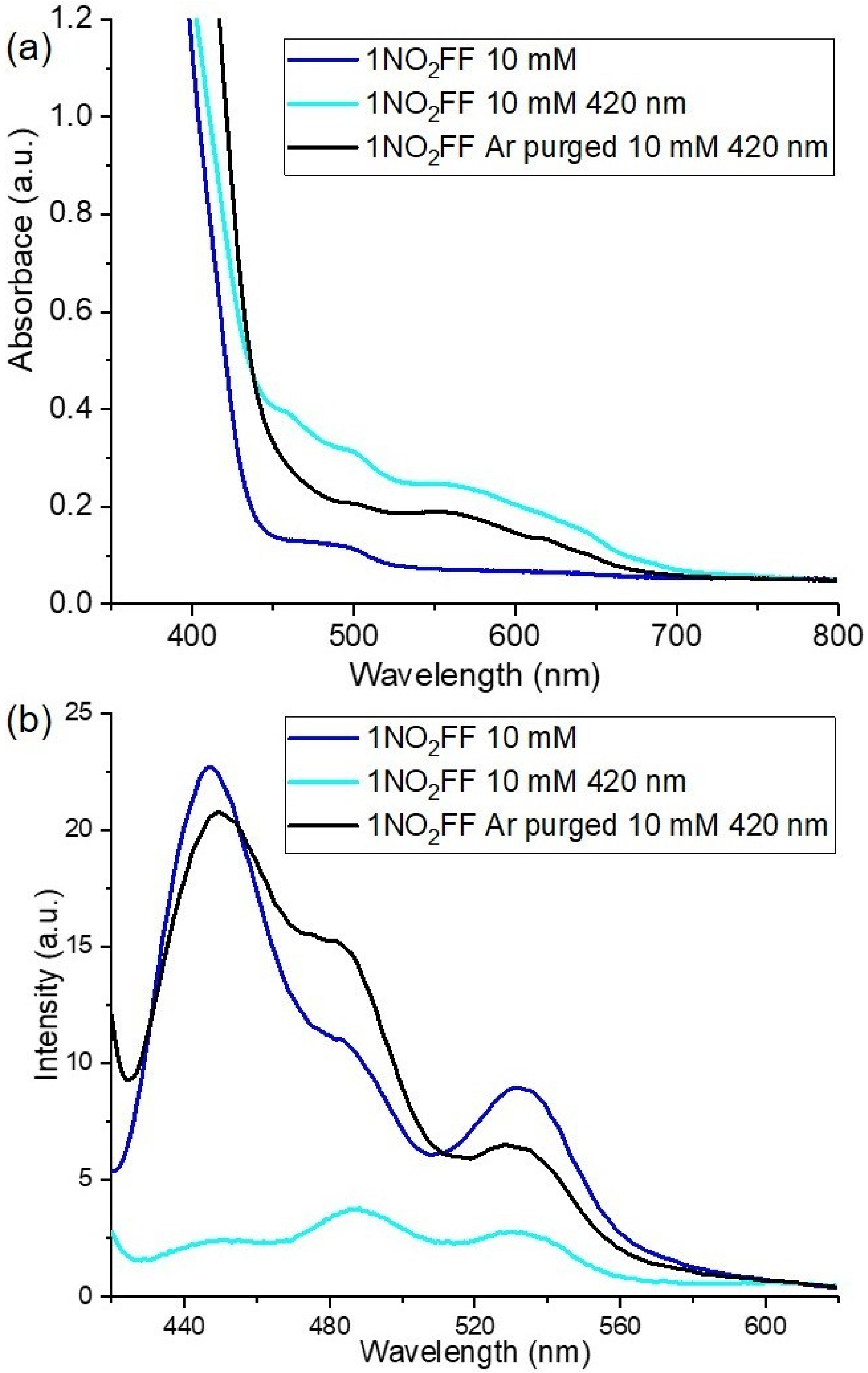

Fig. S17. Comparison of **1NO$_2$FF** before and after irradiation, and Ar-purged **1NO$_2$FF** after irradiation (420 nm, 30 min): (a) UV-vis absorbance and (b) fluorescence emission spectra (λex = 400 nm).

Table S1. Fitting parameters for SAXS data of the single-component peptide solutions

Sample	**1NO$_2$FF**	**1NO$_2$FF** after irradiating 420 nm	**6OMeVF**	**6OMeVF** after irradiating 420 nm
Model	Cylinder + Power law		Power law	
Background (cm^{-1})	0.0032 ± 5.56 × 10^{-5}	0.0041 ± 1.07 × 10^{-4}	0.0081 ± 2.89 × 10^{-5}	0.0077 ± 2.92 × 10^{-5}
A Scale	0.00043 ± 1.86 × 10^{-6}	0.00084 ± 3.32 × 10^{-6}	-	-
A Length (Å)	436 ± 5	211 ± 2	-	-
A Radius (Å)	33.0 ± 0.04	35.8 ± 0.05	-	-
B Scale	1.73 × 10^{-5} ± 1.33 × 10^{-6}	1.23 × 10^{-4} ± 3.69 × 10^{-6}	8.68 × 10^{-7} ± 5.03 × 10^{-8}	1.32 × 10^{-6} ± 7.52 × 10^{-8}
B Power	2.23 ± 0.02	2.11 ± 0.01	2.42 ± 0.01	2.32 ± 0.01
χ^2	5.49	7.69	2.04	1.57

Table S2. Fitting parameters for SAXS data of the multicomponent peptide solutions

Sample	**1NO$_2$FF** + **6OMeVF** 1:1 mixture (yellow)	**1NO$_2$FF** + **6OMeVF** 1:1 mixture (red, after irradiating 420 nm)	**1NO$_2$FF** + **6OMeVF** 1:1 mix (yellow, after irradiating 420 nm and recovered for 14 days)
Model	Cylinder + Power law		
Background (cm^{-1})	0.0070 ± 5.94 × 10^{-5}	0.0082 ± 5.30 × 10^{-5}	0.0077 ± 4.12 × 10^{-5}
A Scale	0.0004 ± 1.72 × 10^{-6}	0.00033 ± 1.74 × 10^{-6}	0.00024 ± 1.43 × 10^{-6}
A Length (Å)	540 ± 7	218 ± 2	230 ± 3
A Radius (Å)	31.5 ± 0.04	31.5 ± 0.05	32.8 ± 0.06
B Scale	2.13 × 10^{-5} ± 2.16 × 10^{-6}	1.43 × 10^{-5} ± 1.40 × 10^{-6}	1.40 × 10^{-5} ± 4.83 × 10^{-7}
B Power	1.98 ± 0.019	2.11 ± 0.018	2.40 ± 0.006
χ^2	2.86	2.60	1.16

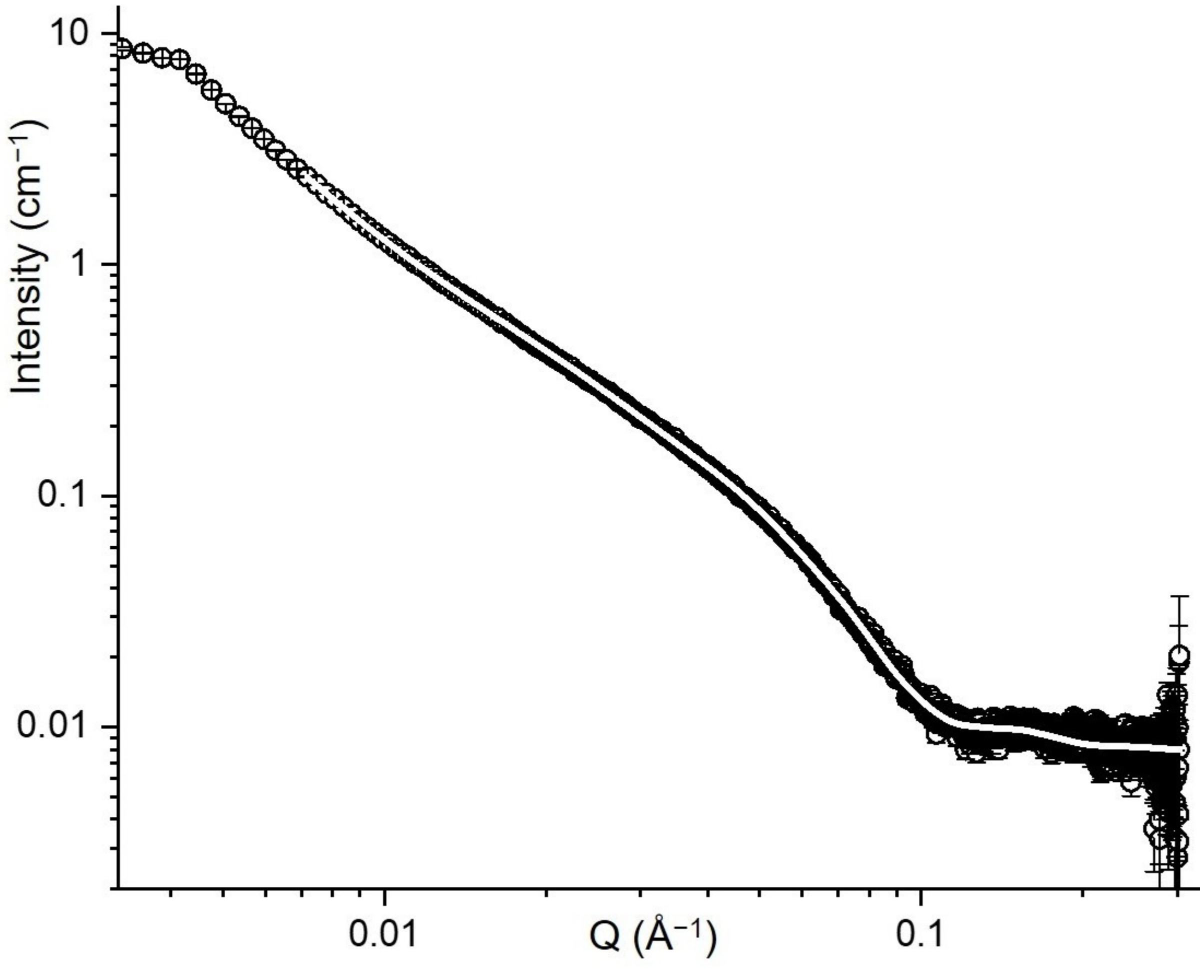

Fig. S18. SAXS profile of the equimolar **$1NO_2FF$** + **6OMeVF** mixture after irradiation at 420 nm and recovery in the dark for 14 d. The data are shown in empty black circles and the fit is shown with solid white line.

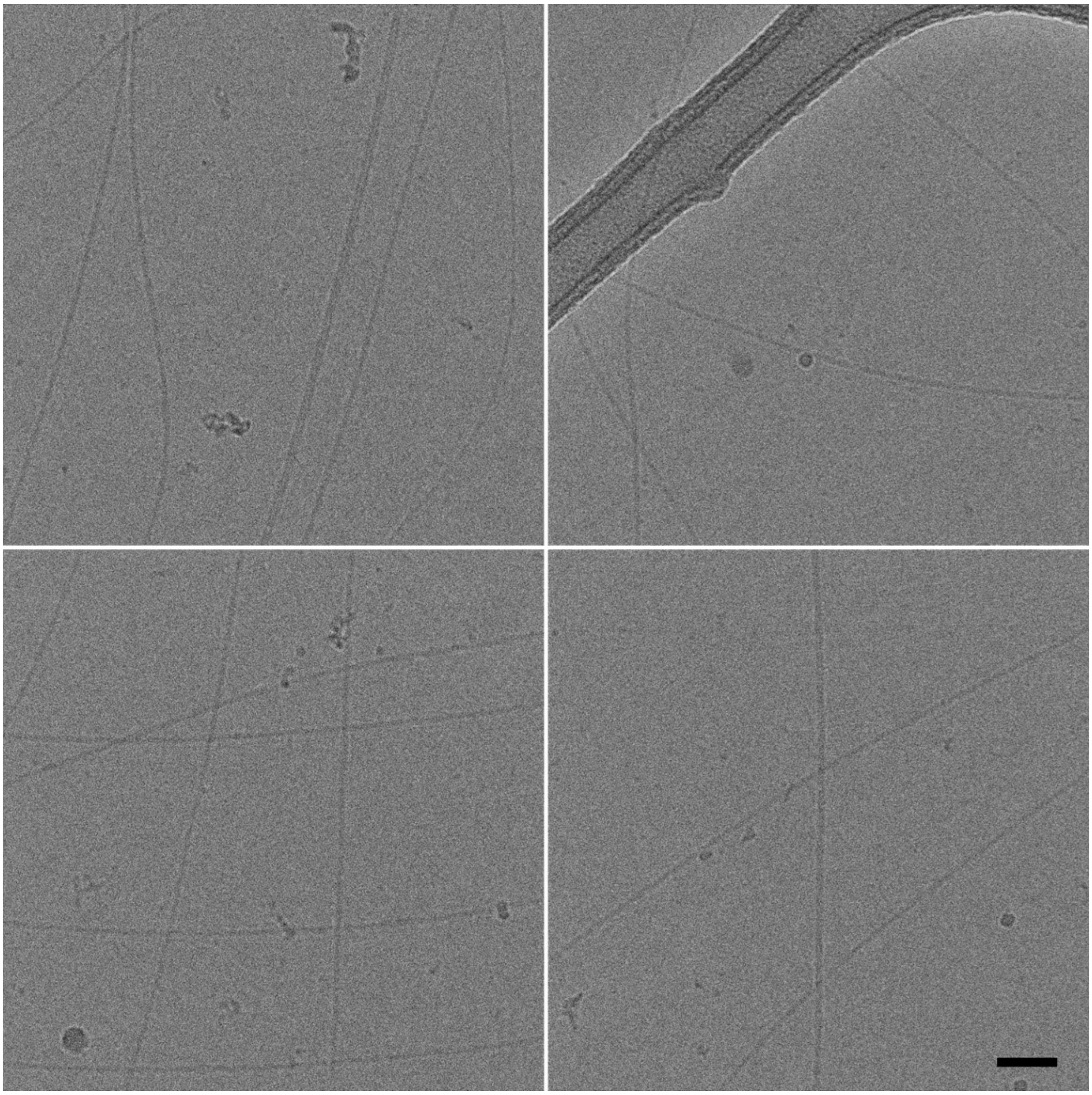

Fig. S19. Cryo-EM images of vitrified solutions of 10 mM **$1NO_2FF$**, prepared under ambient condition. Scale bar = 50 nm (same for all Figures).

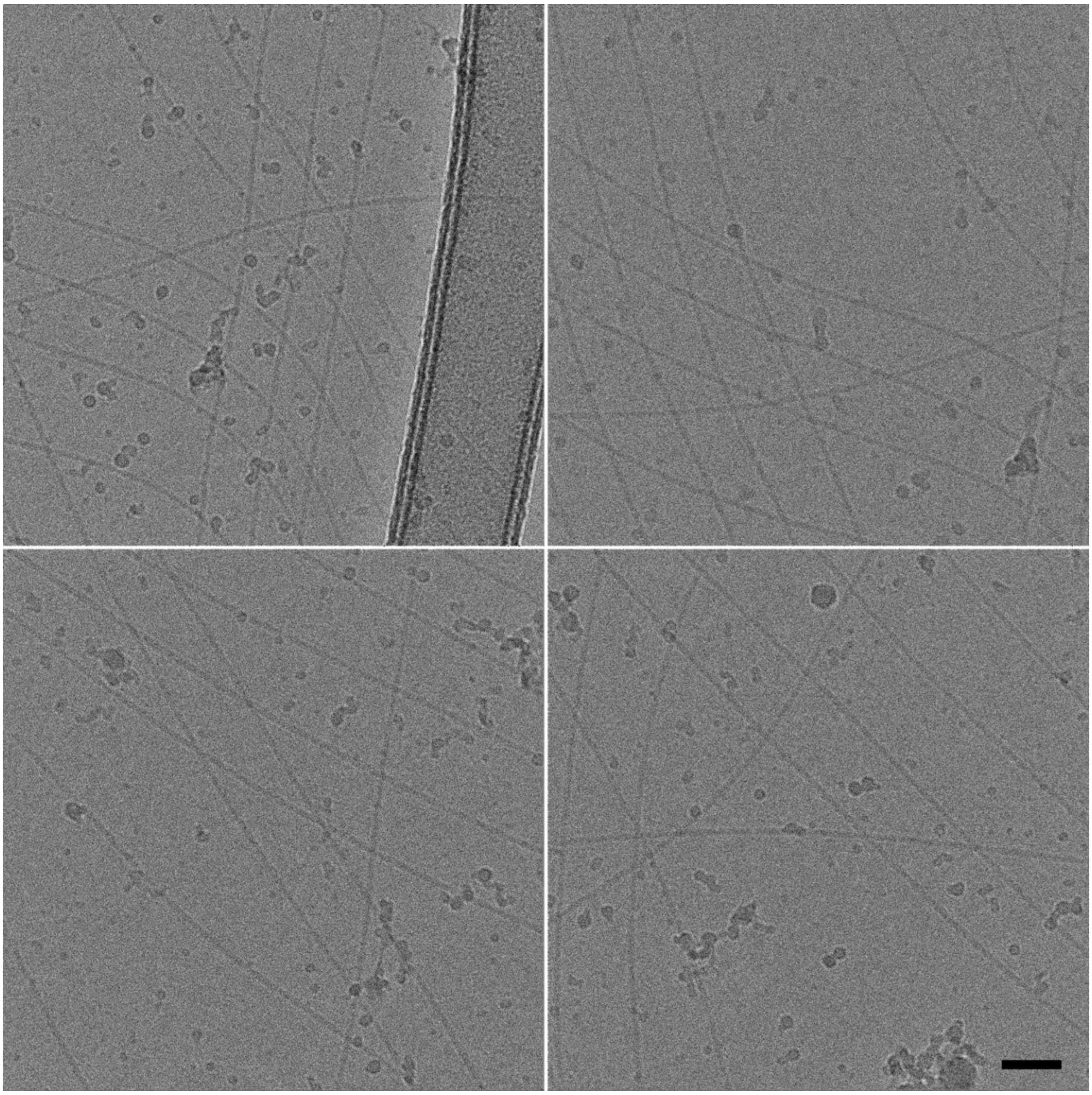

Fig. S20. Cryo-EM images of vitrified solutions of 10 mM **1NO$_2$FF** after stirring for 30 min under solar simulator. Scale bar = 50 nm (same for all Figures). The spherical spots observed in the images are vitrification artefacts, likely from frozen ethane or ice contamination, not an intrinsic sample feature.

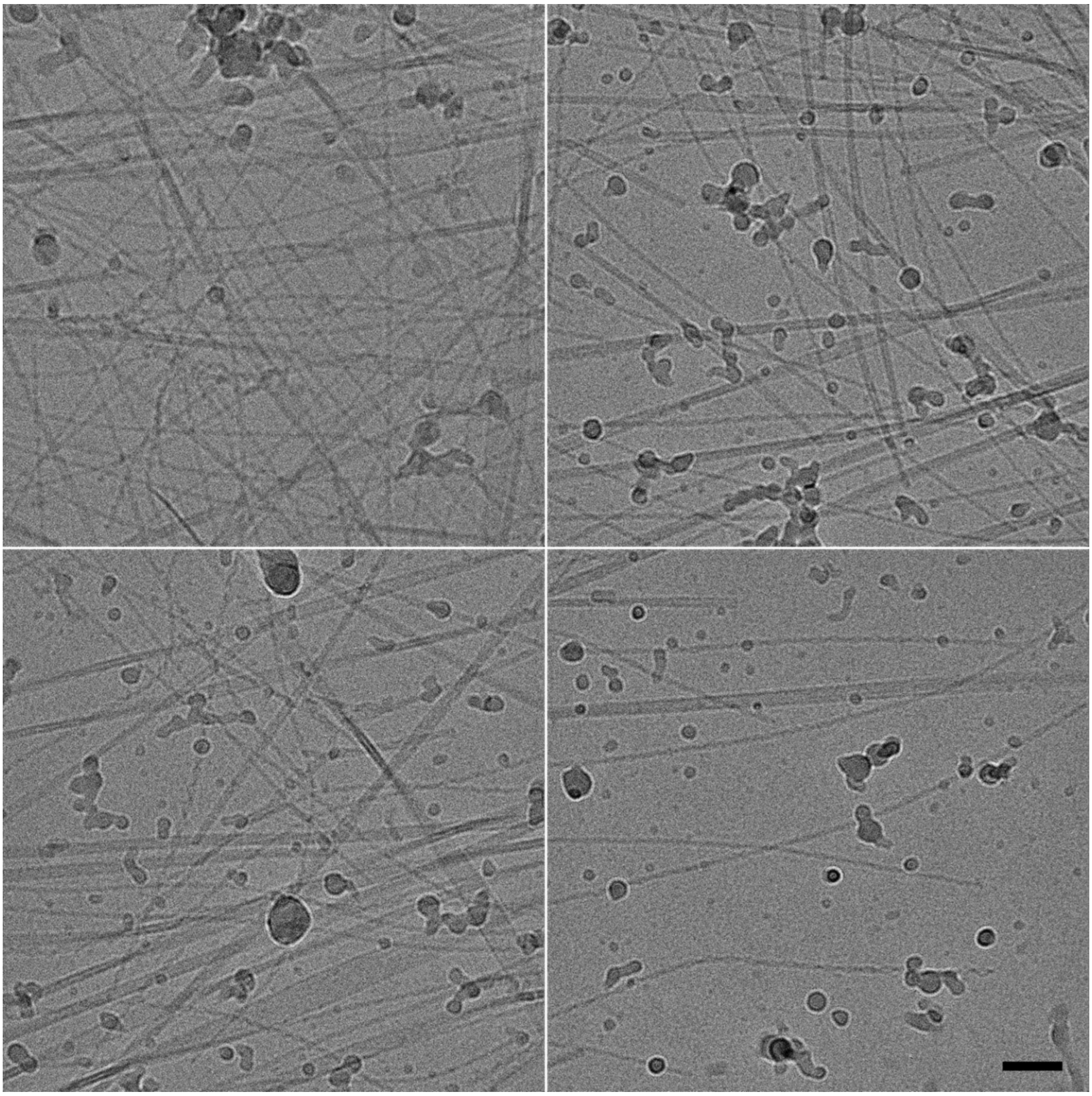

Fig. S21. Cryo-EM images of vitrified solutions of 10 mM **6OMeVF**, prepared under ambient condition. Scale bar = 50 nm (same for all Figures). The spherical spots observed in the images are vitrification artefacts, likely from frozen ethane or ice contamination, not an intrinsic sample feature.

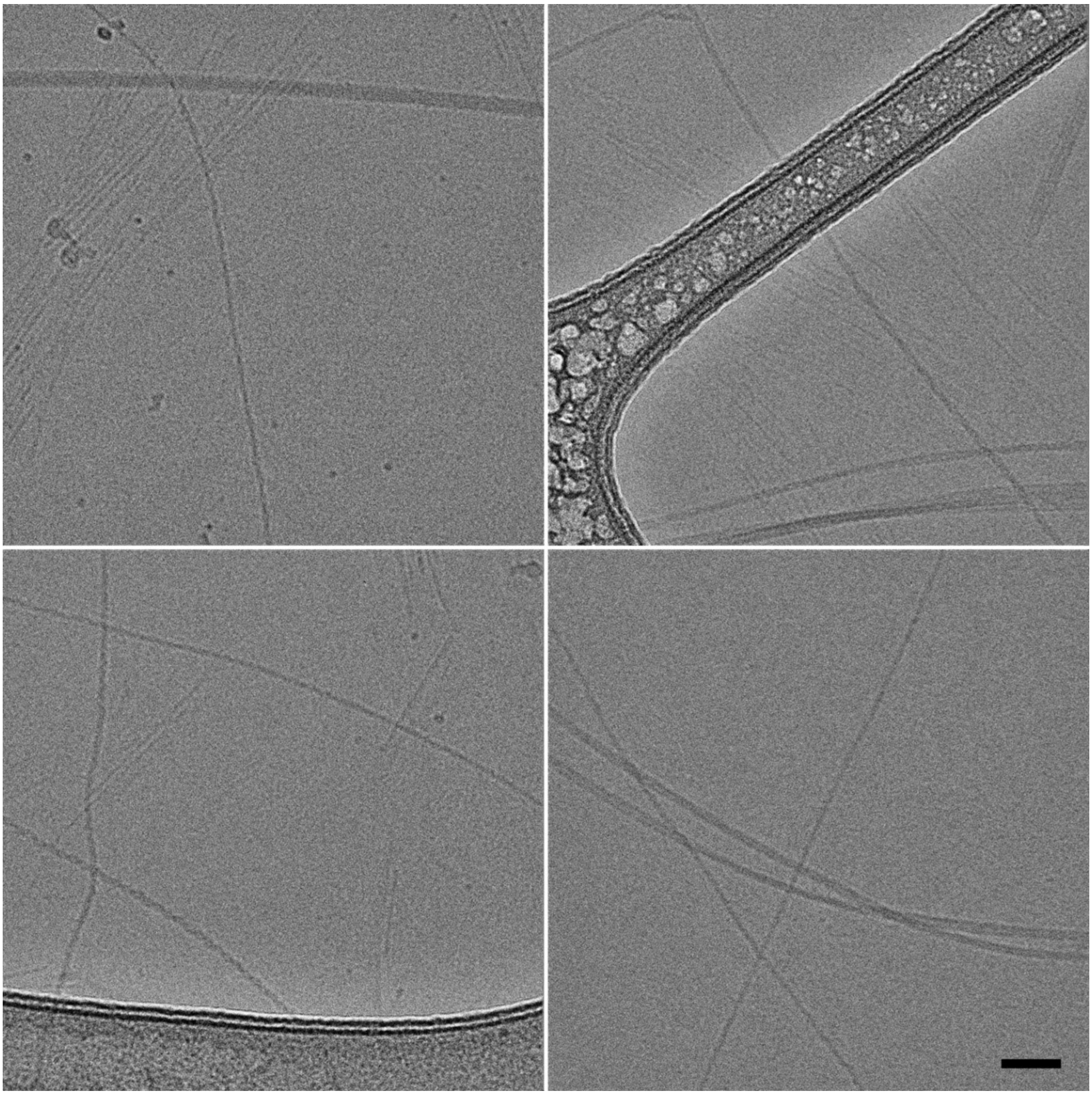

Fig. S22. Cryo-EM images of vitrified solutions of 10 mM **6OMeVF** after stirring for 30 min under solar simulator. Scale bar = 50 nm (same for all Figures).

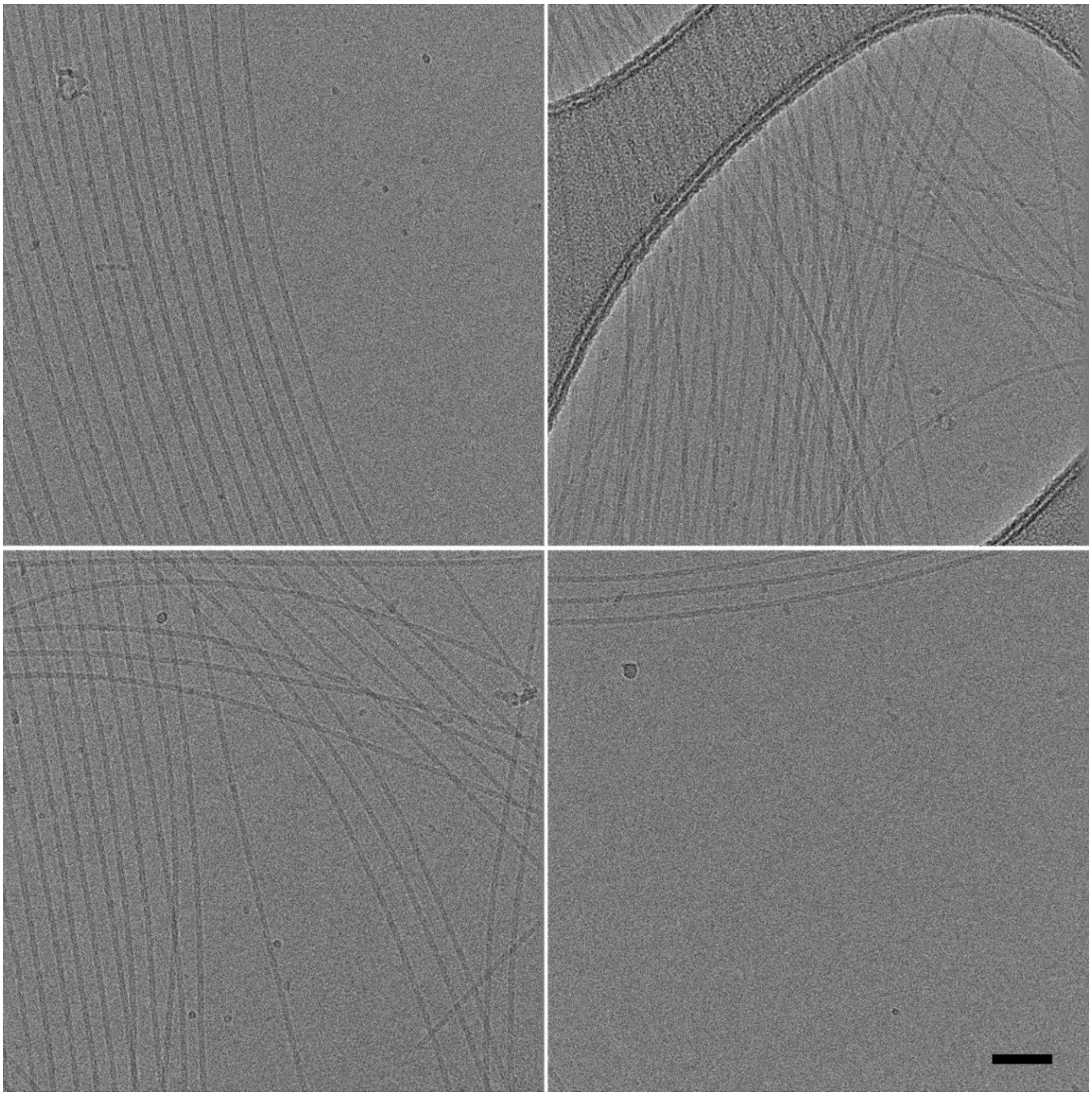

Fig. S23. Cryo-EM images of vitrified solutions of **1NO$_2$FF+6OMeVF** (5 mM each), prepared under ambient condition. Scale bar = 50 nm (same for all Figures).

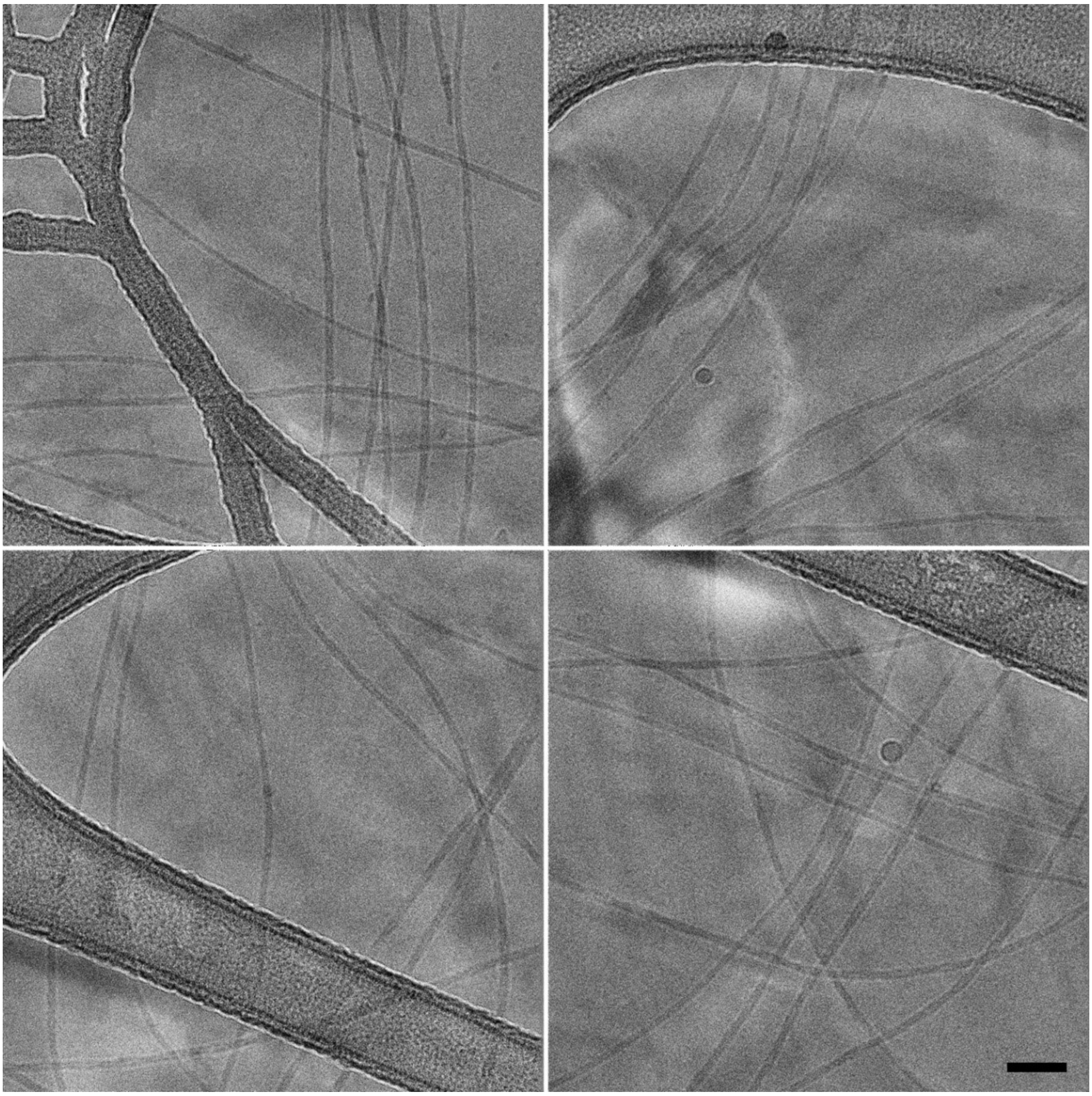

Fig. S24. Cryo-EM images of vitrified solutions of **$1NO_2FF$+6OMeVF** (5 mM each) after stirring for 30 min under solar simulator. Scale bar = 50 nm (same for all Figures).

References

1. K. McAulay, B. Dietrich, H. Su, M. T. Scott, S. Rogers, Y. K. Al-Hilaly, H. Cui, L. C. Serpell, Annela M. Seddon, E. R. Draper and D. J. Adams, *Chem. Sci.*, 2019, **10**, 7801-7806.
2. L. Chen, T. O. McDonald and D. J. Adams, *RSC Adv.*, 2013, **3**, 8714-8720.
3. T. S. Plivelic, A. E. Terry, R. Appio, K. Theodor and K. Klementiev, *AIP Conference Proceedings*, 2019, **2054**.
4. J. Filik, A. W. Ashton, P. C. Y. Chang, P. A. Chater, S. J. Day, M. Drakopoulos, M. W. Gerring, M. L. Hart, O. V. Magdysyuk, S. Michalik, A. Smith, C. C. Tang, N. J. Terrill, M. T. Wharmby and H. Wilhelm, *J. Appl. Crystallogr.*, 2017, **50**, 959-966.
5. SasView - Small Angle Scattering Analysis, https://www.sasview.org/, (accessed 03 Mar, 2026).
